# DNA assembly with error correction on a droplet digital microfluidics platform

**DOI:** 10.1186/s12896-018-0439-9

**Published:** 2018-06-01

**Authors:** Yuliya Khilko, Philip D. Weyman, John I. Glass, Mark D. Adams, Melanie A. McNeil, Peter B. Griffin

**Affiliations:** 1 Stanford Genome Technology Center, Stanford University, 3165 Porter Drive, Palo Alto, CA 94304 USA; 2 Department of Biomedical, Chemical and Materials Engineering, San Jose State University, 1 Washington Sq, San Jose, CA 95192 USA; 3 J. Craig Venter Institute, 4120 Capricorn Lane, La Jolla, CA 92037 USA

**Keywords:** DNA, Gibson assembly, Digital microfluidics, Error correction

## Abstract

**Background:**

Custom synthesized DNA is in high demand for synthetic biology applications. However, current technologies to produce these sequences using assembly from DNA oligonucleotides are costly and labor-intensive. The automation and reduced sample volumes afforded by microfluidic technologies could significantly decrease materials and labor costs associated with DNA synthesis. The purpose of this study was to develop a gene assembly protocol utilizing a digital microfluidic device. Toward this goal, we adapted bench-scale oligonucleotide assembly methods followed by enzymatic error correction to the Mondrian™ digital microfluidic platform.

**Results:**

We optimized Gibson assembly, polymerase chain reaction (PCR), and enzymatic error correction reactions in a single protocol to assemble 12 oligonucleotides into a 339-bp double- stranded DNA sequence encoding part of the human influenza virus hemagglutinin (HA) gene. The reactions were scaled down to 0.6-1.2 μL. Initial microfluidic assembly methods were successful and had an error frequency of approximately 4 errors/kb with errors originating from the original oligonucleotide synthesis. Relative to conventional benchtop procedures, PCR optimization required additional amounts of MgCl_2_, Phusion polymerase, and PEG 8000 to achieve amplification of the assembly and error correction products. After one round of error correction, error frequency was reduced to an average of 1.8 errors kb^− 1^.

**Conclusion:**

We demonstrated that DNA assembly from oligonucleotides and error correction could be completely automated on a digital microfluidic (DMF) platform. The results demonstrate that enzymatic reactions in droplets show a strong dependence on surface interactions, and successful on-chip implementation required supplementation with surfactants, molecular crowding agents, and an excess of enzyme. Enzymatic error correction of assembled fragments improved sequence fidelity by 2-fold, which was a significant improvement but somewhat lower than expected compared to bench-top assays, suggesting an additional capacity for optimization.

**Electronic supplementary material:**

The online version of this article (10.1186/s12896-018-0439-9) contains supplementary material, which is available to authorized users.

## Background

Over the last decade, major research advances in genome sequencing (i.e. “DNA reading”) are slowly being matched by advances in synthetic biology (i.e. “DNA writing”) [1, 2]. Rapid advances in synthetic biology are fueling a demand for synthetic DNA that will only increase in the future. However, the ability to synthesize long DNA molecules in a short period of time without significant expense remains one of the main challenges in synthetic biology [3–5].

Gene synthesis is a costly and labor-intensive process. The cost of synthetic DNA is directly related to the cost of oligonucleotides, and a large amount of hands-on labor required for conventional oligonucleotide-based gene assembly is also a significant cost [6–8]. The cheapest oligonucleotides that can be purchased from commercial suppliers are usually unpurified and contain errors. Thus, the genes assembled from the unpurified oligonucleotides must be sequence verified to find a correct assembly. Implementation of an enzymatic error correction step greatly improves the sequence fidelity of the assemblies, which reduces the number of clones that have to be individually cloned and sequence verified [7, 9, 10]. Unfortunately, this extra error correction step also significantly increases the hands-on time required to complete assembly. Integration of digital microfluidics into DNA assembly coupled with error correction can potentially ease this labor burden by allowing for a “set up and walk away” approach to managing the entire process.

Digital microfluidics (DMF) is a technology based on the electrowetting phenomenon. The phenomenon describes a change of surface tension at a solid/liquid/gas interface by application of an electric field [11, 12]. The voltage applied to the electrodes lowers the surface tension, which leads to a reduction of the contact angle and increases the wettability of the surface. Consequently, the liquid spreads over the surface where the voltage is applied. Thus, a hydrophobic surface becomes hydrophilic. By the application of a voltage on a dielectric surface, the liquids can be transported over the surface of a microfluidic cartridge.

**Fig. 1:**
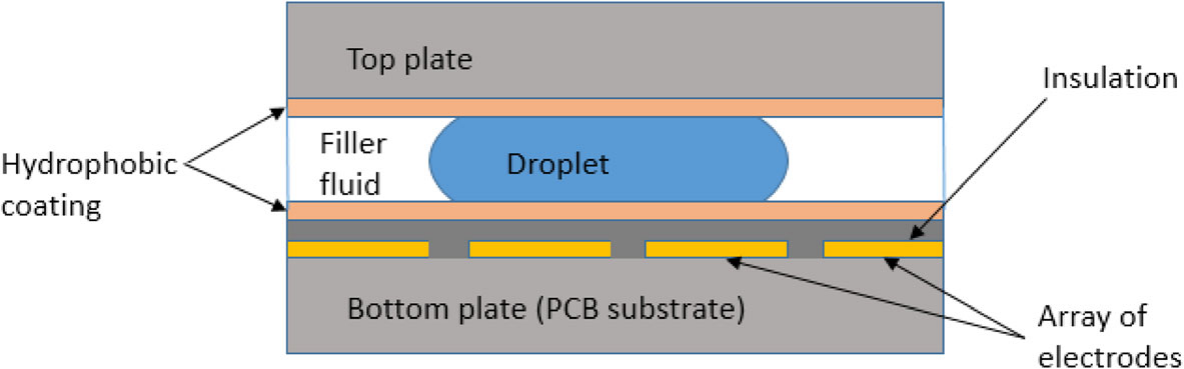
A cross-section of an EWOD cartridge

In electrowetting on dielectric (EWOD) devices, a droplet is sandwiched between two hydrophobic plates and the remaining volume is filled with immiscible liquid, for example, a silicone oil (Fig. 1). The oil prevents evaporation of the aqueous droplets and facilitates transport. The bottom plate is the array of electrodes, which can locally control the surface tension by the application of a voltage. Digital microfluidic devices are completely programmable and do not require any pumps or valves to move the liquids. A cartridge can be inserted into a micro-controller that is operated by a software program [13–15]. The program turns the voltage on and off at certain electrodes, so the droplets can be directed anywhere on a chip. They can also be dispensed, transported, split, fused, mixed and held in certain regions.

Digital microfluidic devices are applicable for gene assembly because DNA is typically handled in microliter amounts. The microfluidic devices are capable of generating droplets in the microliter to picoliter range [16, 17]. The microliter droplets act like reaction and transport vessels. The ability to program liquid handling operations such as dispense, transport, merge, mix, and split allows a researcher to automate and simplify the gene assembly process. Time-consuming steps like pipetting, transferring reagents, tube labeling, incubation at certain temperatures, and thermocycling can be replaced and performed by programmable droplet generation and routing over heater bars underneath the microfluidic cartridge. The sequential reactions can be performed on a single microfluidic cartridge without any human intervention [18]. Software automation programs can be designed to conduct multiple experiments in parallel. Since the devices are fully automated, the sources of human errors and labor costs can be greatly reduced. In addition, DNA assembly programs can be distributed between laboratories, so that scientists can share robust protocols.

Because DNA assembly and error correction reactions require the use of expensive enzymes, scaling down to smaller reaction volumes reduces reagent expenses. Due to the large surface-to-volume ratio, micro-droplet reactors have high heat and mass transfer rates. This makes it possible to increase kinetics and speed up reactions [13, 17, 19]. Integration of effective error correction procedures could permit DNA assembly on a single microfluidic cartridge without the need for expensive and lengthy sequence verification.

A number of DNA assembly protocols have been developed to date (Table 1). For the scope of this study, only assembly methods from oligonucleotides will be discussed. The most popular gene construction methods for microfluidic applications are polymerase-based and endonuclease-based assembly. Both approaches utilize oligonucleotides as DNA building blocks [20]. The polymerase-based assembly method utilizes the same approach as PCR [21–24]), but instead of using forward and reverse primers, oligonucleotides overlap and serve as templates for a complementary strand. The oligonucleotides are designed to be either a part of the top or the bottom DNA strand. In the first assembly cycle, oligonucleotides overlap partially, and the polymerase extends the complementary strand in a 5′ to 3′ direction. In the next cycle, the double-stranded DNA pieces are separated and hybridized to other oligonucleotides or assembled single-stranded fragments. The process of denaturation, annealing, and extension is repeated until the desired sequence has been built [25].

**Table 1:** Summary of microfluidic assembly methods

Authors	Type of microfluidics	Assembly method	Assembly size	Error rates
Kong et al. [22]	Droplet	Polymerase-based (PCA)	500 –1000 bp	1.78 errors/kb
Huang et al. [21]	Microchannel	Polymerase-based (PCA)	760 bp	4.1 errors/kb
Quan et al. [23]	Microarray	Polymerase-based (PCA)	500 – 1000 bp	1.9 errors/kb
Tian et al. [24]	Microarray	Polymerase-based (PAM)	14,500 bp	2.2 errors/kb
Linshiz et al. [48]	Chanel	Exonuclease-based (Gibson) and polymerase-based (IHDC)	754 bp	N/A
Shih et al. [49]	Combined digital and droplet microfluidics	Exonuclease-based assembly of dsDNA (Gibson)	2100 bp	N/A
Tangen et al. [50]	Droplets	Polymerase-based (PCA) and Exonuclease-based (Gibson)	525 bp	N/A
Ben-Yehezkel et al. [18]	Digital microfluidics	Polymerase-based (POP)	800 bp	2.22 errors/kb

The studies shown in Table 1 utilized different types of microfluidics to perform DNA assembly. Among those studies, only the work by Ben-Yehezkel et al. was conducted using digital microfluidics, the same type of microfluidics utilized in the present study. The group developed an innovation on the polymerase-based assembly method called programmable order polymerization (POP). The method was first successfully automated on the Mondrian™ microfluidic device. The assembly reaction proceeded to assemble the sequence from the inside out. In each of four phases, a double-stranded DNA (dsDNA) fragment was extended by a pair of oligonucleotides such that one oligonucleotide bound to each end of the sequence. Multiple rounds of thermocycling for each phase with an oligonucleotide pair ensured that most of the product was extended in each step. The group reported an error rate of 1 in 450 bp (2.22 errors kb^− 1^) for their assembly method, and the errors were identified as substitutions [18].

Whereas Ben-Yehezkel used four separate rounds of DNA polymerization to sequentially extend and assemble two DNA fragments in each round, we used a single “one-pot” assembly of 12 fragments in a single droplet and successfully assembled the complete sequence. In addition, we performed a round of error correction on the digital microfluidic device. At some point, a large number of DNA fragments in a one-pot reaction would lead to miss-hybridization, so it is interesting to contemplate a process that combines both these methods in a DNA assembly reaction to reduce the assembly time and errors even further.

In summary, the one-pot assembly method used in this manuscript is a one-step isothermal Gibson assembly developed at the J. Craig Venter Institute ([26]) and is distinctly different in process from the work described by Ben-Yehezkel et al. With this technique, double- or single-stranded DNA pieces are joined into longer fragments by three enzymes: T5 exonuclease, DNA polymerase, and Taq DNA ligase. The reagents are incubated at 50 °C for 0.5-1 h after which the assembled product is typically amplified by PCR [27]. Published protocols use multiple rounds of error correction after assembly and PCR to decrease the incidence of errors originating from the oligonucleotides [28]. Gibson assembly has been used successfully to assemble entire genes (1.5-1.7 kb) in a single step, and this method is arguably the most efficient to assemble genes from multiple oligonucleotides [28]. Using Gibson assembly, higher numbers of oligonucleotides can be assembled in a single reaction than by PCR assembly. For this reason, we decided to implement Gibson assembly on a DMF device.

**Fig. 2:**
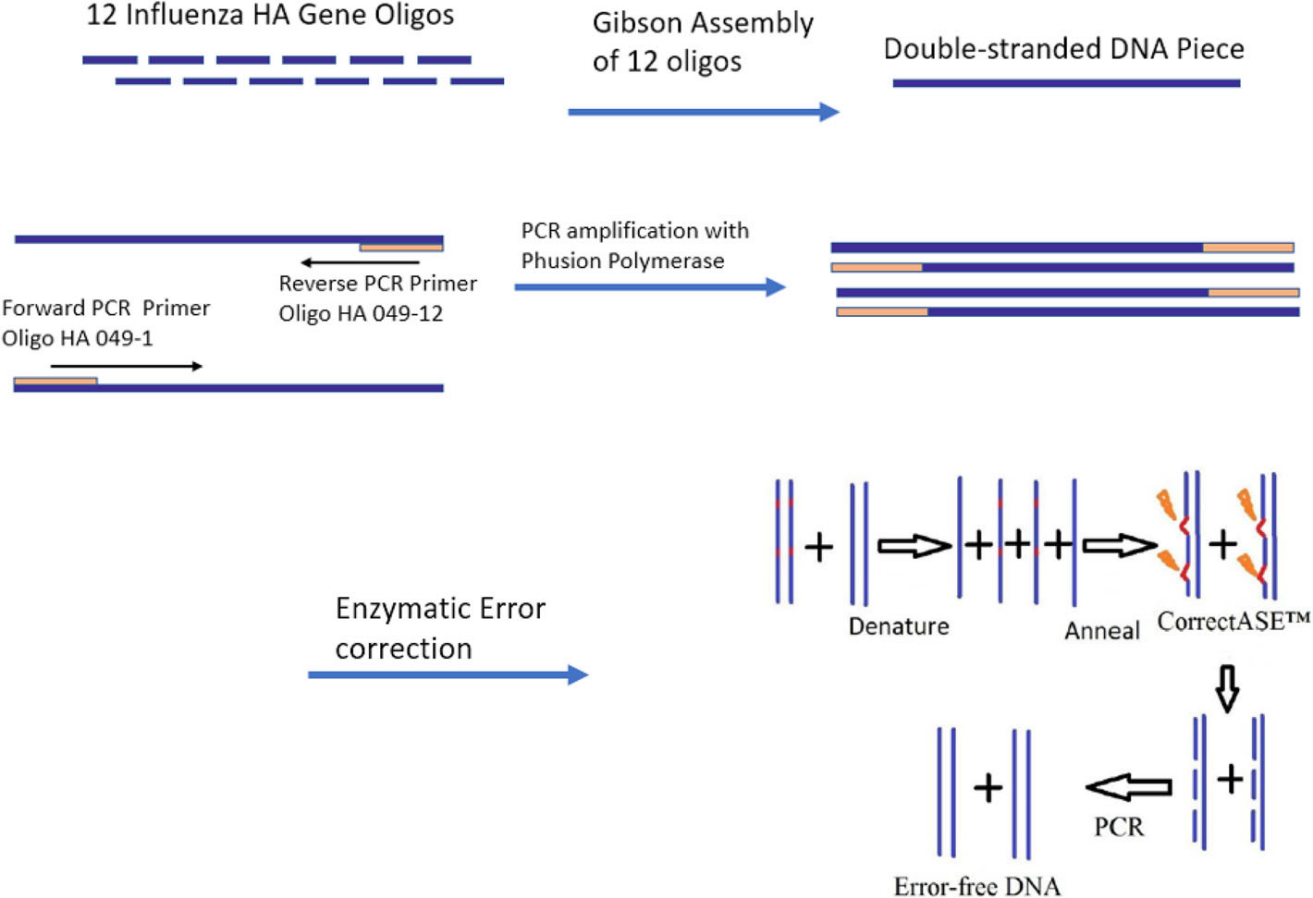
A schematic of influenza HA gene assembly on DMF. This diagram shows the steps of the process which were all performed sequentially on a microfluidic cartridge. The steps are Gibson assembly of 12 oligonucleotides, PCR amplification of a double-stranded DNA piece, error removal using an error correction enzyme, and PCR amplification of corrected sequences

To design a DNA assembly protocol for a programmable digital microfluidic device, we developed a process consisting of three major parts (Fig. 2). First, DNA oligonucleotides were assembled into a double-stranded DNA fragment. Second, the assembly was amplified by PCR, and third, errors from the original oligonucleotides were removed. We used Sanger DNA sequencing of the recovered, error-corrected products to verify the efficiency of the error correction process and develop an efficient DNA assembly and error correction protocol. The ultimate goal is to design a reliable and cost-effective DNA assembly protocol that will be widely applicable in biological research.

## Methods

### DNA sequences and oligonucleotides

**Fig. 3:**
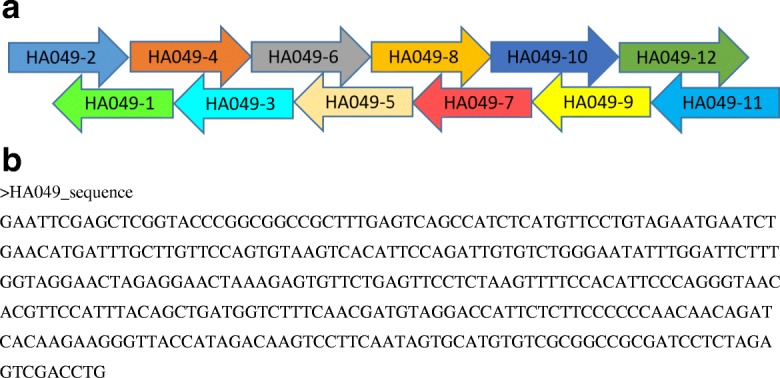
Oligonucleotide alignment for the 339-bp test assembly sequence. **a** Arrangement of DNA oligonucleotides used for assembly of the HA049 sequence. **b** FASTA formatted HA049 sequence

Our 339-bp test assembly sequence comprised a partial sequence from the human influenza virus H9N2 hemagglutinin (HA) gene (283-bp, nt 211-493 of the HA coding region) flanked on each side by 8-bp NotI restriction endonuclease sites and 20-bp homology regions to the pUC19 cloning vector. The 339-bp fragment was divided into 12 overlapping oligonucleotides (See Table 2). The final assembled test sequence is listed in Fig. 3.

**Table 2:** Primers used in this study

Primer Name	Primer Sequence (5′- > 3′)
Puc-049cloning-R	CCGGGTACCGAGCTCGAATTCACTG
Puc-049cloning-F	GATCCTCTAGAGTCGACCTGCAGGC
Puc19-5’F	TCCCAGTCACGACGTTGTAAAACGAC
HA049-1	CAGGTCGACTCTAGAGGATCGCGGCCGCGACACATGCACTATTGAAGGACTT
HA049-2	AGATCACAAGAAGGGTTACCATAGACAAGTCCTTCAATAGTGCATGTGTCGC
HA049-3	GTCTATGGTAACCCTTCTTGTGATCTGTTGTTGGGGGGAAGAGAATGGTCCT
HA049-4	TACAGCTGATGGTCTTTCAACGATGTAGGACCATTCTCTTCCCCCCAACAAC
HA049-5	ACATCGTTGAAAGACCATCAGCTGTAAATGGAACGTGTTACCCTGGGAATGT
HA049-6	GTGTTCTGAGTTCCTCTAAGTTTTCCACATTCCCAGGGTAACACGTTCCATT
HA049-7	GGAAAACTTAGAGGAACTCAGAACACTCTTTAGTTCCTCTAGTTCCTACCAA
HA049-8	ATTGTGTCTGGGAATATTTGGATTCTTTGGTAGGAACTAGAGGAACTAAAGA
HA049-9	AGAATCCAAATATTCCCAGACACAATCTGGAATGTGACTTACACTGGAACAA
HA049-10	GTAGAATGAATCTGAACATGATTTGCTTGTTCCAGTGTAAGTCACATTCCAG
HA049-11	GCAAATCATGTTCAGATTCATTCTACAGGAACATGAGATGGCTGACTCAAAG
HA049-12	GAATTCGAGCTCGGTACCCGGCGGCCGCTTTGAGTCAGCCATCTCATGTTCCT

### Mondrian™ digital microfluidics (DMF) device

The main goal of this study was to develop a robust and reliable DNA assembly and error correction protocol for the Mondrian™ DMF device (Illumina, Inc.). The whole protocol involved four consecutive enzymatic reactions. Prior to incorporating the four enzymatic steps of the gene assembly in a complete protocol, each enzymatic step was optimized individually. All liquid handling operations were programmed using the Application Development Environment software (Illumina, Inc.).

**Fig. 4:**
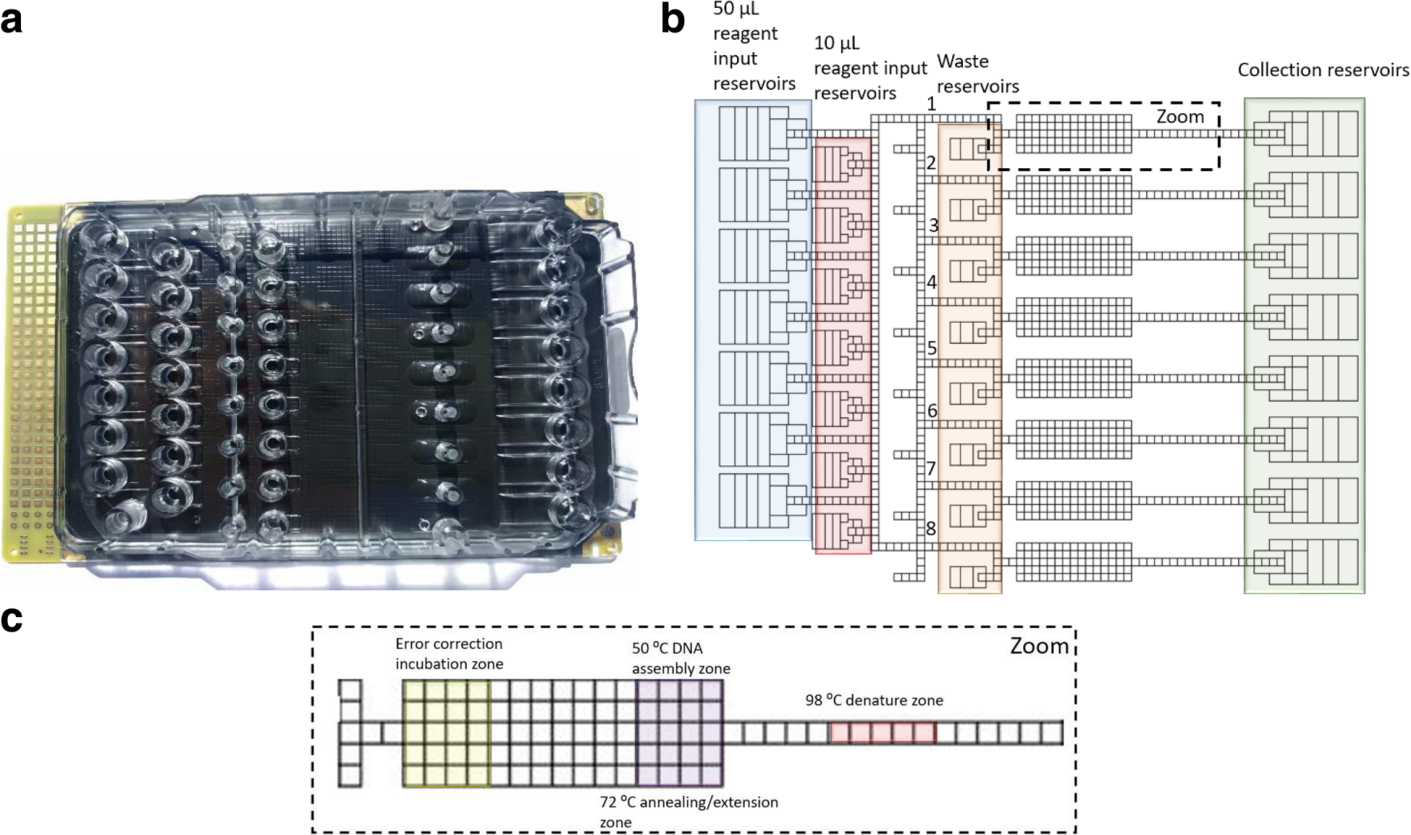
The Mondrian™ microfluidic cartridge. **a** Image of the cartridge. **b** Diagram of the cartridge electrode paths. This diagram of the chip comes from a screenshot of an ADE software. There are 50 μL reservoirs highlighted in blue, 10 μL reservoirs highlighted in red. Orange reservoirs were used to hold waste. Green reservoirs were used to collect final products. The configuration of the chip allowed to perform 8 reactions in parallel. **c** Magnified image of one lane of the microfluidic cartridge where the reactions were performed. The area highlighted in yellow was used for error correction reaction. The area highlighted in purple was used for Gibson assembly and PCR annealing/extension. The area highlighted in red was used for DNA denaturation during PCR and error correction pretreatment

The Mondrian™ microfluidic system included a microcontroller that was connected to a computer and digital microfluidic cartridges that were inserted into the device. To observe the behavior of the droplets, a digital camera was mounted above the cartridge to produce a magnified image of the DMF cartridge onto a computer screen. The Mondrian™ cartridge that was used in the experiments (Fig. 4a), consisted of two plates, a plastic top plate, and a printed circuit board (PCB) substrate. The area between the plates was filled with a 2 cSt silicone oil. As seen in Fig. 4b, the configuration of the DMF cartridge allowed eight processes to be performed in parallel. The reagents were loaded through 50 μL or 10 μL ports on the cartridge top plate, and the samples were withdrawn through other ports. There were also seven reservoirs dedicated for the collection of waste droplets. The microfluidic cartridge had three heater bars that contacted the back of the PCB, which were used to set temperatures for the enzymatic reactions. Additionally, an area of the cartridge could be cooled down with a Peltier device. Fig. 4c shows a close-up view of one lane with three different temperature zones, which were maintained during reactions using the heaters and the cooler. The device was operated by the Application Development Environment (ADE) software. Prior to each experiment, a program was designed to direct droplets through the liquid handling operations. The device was operated at voltages between 90 V and 300 V and at a frequency of 30 Hz.

**Fig. 5:**
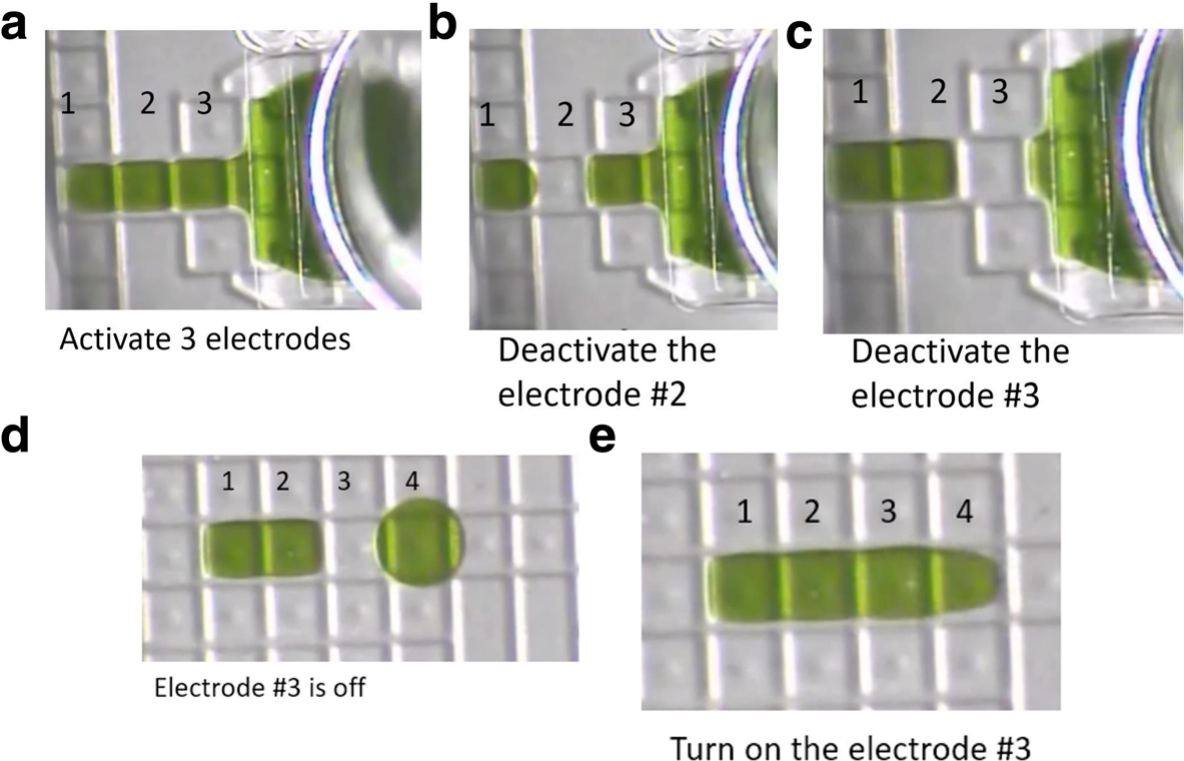
Generation of 0.3, 0.6, and 1.2 μL droplets on DMF. **a** Stretching the liquid over three electrodes. **b** Generation of 0.3 μL droplet. **c** Generation of 0.6 μL droplet. **d** Two 0.6 μL droplets separated by 1 electrode. **e** 1.2 μL droplet resulted from merging two 0.6 μL droplets

The liquid volumes of 0.3, 0.6, and 1.2 μL were generated and manipulated on the microfluidic cartridge. To dispense a 0.3 μL or 0.6 μL droplet, three electrodes adjacent to the reagent input port were activated, which caused the liquid to spread over three electrodes (Fig. 5a). The electrode #2 was switched off to obtain a 0.3 μL droplet (Fig. 5b). The double 0.6 μL droplet was dispensed by turning off the electrode #3 (Fig. 5c). To create a 1.2 μL droplet, two 0.6 μL droplets are brought next to each other and separated by one inactive electrode as shown in Fig. 5d. Then the electrode between the two 0.6 μL droplets was turned on, merging both into one 1.2 μL droplet (Fig. 5e). Refer to Additional file 1: Video 1 to see the liquid handling operation described here. Materials to see all liquid handling operations used in present work.

### Optimization of microfluidic PCR

Each on-chip PCR reaction contained 1X HF Phusion buffer, detergent-free (Thermo Fisher Scientific), 0.8 μM forward and reverse primers, 1.75 ng/μL HA-049 DNA template (plasmid-cloned HA-049 sequence), 0.1 U μL^− 1^ Phusion polymerase (Thermo Fisher Scientific). The reactions were set up to allow the addition of 1 mM MgCl_2_, 1.25 mM PEG 8000, 0.2 mM NAD, 2 mM DTT to the reaction mixture or the combination of 1.25 mM PEG 8000 and 1 mM MgCl_2_, 0.2 mM NAD, and 2 mM DTT. Final concentrations in the reaction droplets are given for all reagents.

An automation program for the DMF was developed to perform microfluidic PCR amplification experiments. The reactions were carried out in 1.2 μL droplets. The droplets were brought to the PCR area of the chip, which consisted of two temperature zones. The denaturation zone was set to 98 °C, and the annealing/extension zone was set to 72 °C.

The thermocycling was performed at a reduced voltage (90 V), which eliminated nonspecific adsorption of polymerase on the microfluidic surface and reduced spurious bubble formation at high temperatures [18, 27, 29–32]. An initial denaturation was performed by moving the droplets to the 98 °C zone where they were held for 30 s. Then thirty PCR cycles were performed by cycling the droplets from 98 °C to 72 °C at 1.5 s/electrode, and from 72 °C to 98 °C at 1 s/electrode. Annealing/extension of the droplets was done by switching on/off voltage of the area of three electrodes every half of a second for 20 s, and denaturation was performed by holding the droplets at 98 °C for 10 s. After 30 cycles of PCR, DNA was held for 10 min at 72 °C to allow a final extension. Then, the voltage was switched back to 300 V, so that the samples could be transported to the collection reservoirs.

### Optimization of microfluidic Gibson assembly

In each experiment, 50 μL master mixes were made from fresh reagents and prepared according to previously published Gibson Assembly protocols [33] with additional modifications described below. Assembly, oligonucleotide, and PCR master mixes were prepared at 2X concentrations such that once two equal size droplets were merged, the final enzyme mixtures would be at the correct 1X concentration. The oligo master mix containing a mixture of all oligonucleotides was prepared by diluting a 1 μM stock solution in DI water containing 0.01% Tween 20. The surfactant was a necessary component to reduce the surface tension, which facilitated droplet dispensing and movement. The amount of surfactant required was determined for each master mix. The enzymes suspended in storage buffers contained stabilizers. It was observed that the droplets with enzyme solutions were easily dispensed and manipulated on a cartridge without any additional surfactant. Thus, the assembly master mix and PCR master mix did not contain Tween 20. The final (1X) concentrations of reagents in the assembly reaction were 1X isothermal (iso) buffer, 0.05 U μL^− 1^ of Phusion polymerase, 4 U μL^− 1^ DNA ligase, 0.08 U μL^− 1^ T5 exonuclease, and 250 nM oligonucleotides.

To perform DNA assembly experiments, an automation program was created. The temperature in the assembly area was set to 50 °C. Next, 0.3 μL droplets containing oligonucleotides were dispensed. The droplets were transported to a waiting area where they were held while another dispenser generated 2X Gibson master mix droplets. The oligonucleotide and Gibson master mix droplets were merged to obtain a double 0.6 μL droplet and brought to the assembly area where they were incubated for 15-60 min at 50 °C. To ensure adequate mixing, the droplets were moved up and down over 4 electrodes. When the reaction was finished, the assembly droplets were merged with 0.6 μL PCR droplets, so the total volume of each droplet became 1.2 μL. The polymerase chain reaction was performed as described above. After PCR, the products were diluted. To perform dilutions, a dispenser containing DI water and 0.05% Tween 20 generated 0.6 μL droplets. Then, the droplets were merged with the assembly droplets, mixed, and split into two equal size droplets. This step was iterated to achieve the desired dilutions. When assembly time was variable, 0.6 μL droplets containing both the oligonucleotides and Gibson assembly reagents were held in a waiting area, and two droplets were moved to the assembly incubation zone in 15 min increments. This way, each condition was tested twice in two different experimental droplets.

### Optimization of enzymatic error correction

The optimization of enzymatic error correction step was performed using a mix of two equal molar amounts of 339-bp PCR products. Sequences were amplified from two DNA templates. The first template had a completely correct sequence and the other had a mutation approximately in the middle of the 339-bp sequence. If the error correction reaction was successful, two DNA bands were visualized on an agarose gel corresponding to the original size (339-bp) and the cleaved size (approximately 170-bp). In case of a failure, only one 339-bp band was visible. Preliminary experiments demonstrated that a microfluidic error correction reaction with standard benchtop reagents was not successful due microfluidic surface interactions and nonspecific adsorption of CorrectASE™. To test the hypothesis of CorrectASE™ adsorption on the droplet oil/water interface, the mix of DNA was treated with additional reagents. The reactions were performed with extra CorrectASE™, 0.01% Tween 20, 1.25 mM PEG 8000, and 2.5 mM MgCl_2_ to determine which could improve reaction performance.

### Protocol for DNA assembly with error correction

The protocol consisted of four consecutive enzymatic reactions. The process started with Gibson assembly that was carried out for 60 min. Then, the assembly products were amplified in 30 PCR cycles. Next, DNA was treated with CorrectASE™ for 60 min. The error correction products were amplified in a second PCR. According to this protocol, the final concentrations of reagents in Gibson assembly reaction were 1X isothermal (iso) buffer, 0.05 U μL^− 1^ of Phusion polymerase (Thermo Fisher Scientific), 4 U μL^− 1^ DNA ligase (NEB), 0.08 U μL^− 1^ T5 exonuclease (NEB), and 50 nM oligonucleotides (IDT DNA). After assembly, the product was diluted with 0.01% Tween 20 (Sigma Aldrich) by 8-fold. Diluted assemblies were merged with equal size droplets of PCR master mixes to achieve 0.1 U μL^− 1^ Phusion polymerase (Thermo Fisher Scientific), 1X HF detergent-free buffer (Thermo Fisher Scientific), 0.25 mM of each dNTP (Thermo Fisher Scientific), 0.8 μM of forward and reverse primers (IDT DNA, 0.625 mM PEG 8000 (Sigma), 0.5 mM MgCl_2_ (Thermo Fisher Scientific) in the reactions. After amplification, two of the eight droplets were recovered from the chip, and the rest of the droplets were diluted by 2-fold with 0.01% Tween 20 solution to continue to the error correction step.

The EC denature/anneal step of the protocol was implemented to expose the errors in DNA sequence for further CorrectASE™ treatment. During the denaturation step, DNA was diluted to 20-25 ng μL^− 1^ in 1X CorrectASE™ buffer and incubated at 98 °C for 2 min, 25 °C for 5 min, and 37 °C for 5 min. Then, the droplets were merged with CorrectASE™ master mix to a final concentration of 2X CorrectASE™ (Invitrogen), 0.01% Tween 20 (Sigma Aldrich), and 2.5 mM PEG 8000 (Sigma). The master mixes contained double amounts of reagents to obtain 1X concentration after the equal size droplets were merged. The reagents were loaded on a DMF cartridge into dedicated dispensers as prescribed by the automation program. All master mixes except CorrectASE™ were loaded on the cartridge at the beginning of the process. To ensure that the enzyme stayed active, CorrectASE™ was loaded into the dispenser three minutes before it was to be used by the program. At the end of the process, all droplets were collected in 20 μL water containing 0.05% Tween 20 and retrieved from the device manually.

### Cloning and sequencing of assembled and amplified products

Recovered products were brought up to 50 μL in water and an equal volume of Agencourt AMPure XP beads (Beckman Coulter) was added and mixed. After 5 min incubation to bind DNA to the beads, the tube was placed on a magnet and allowed to settle for 5 min. The supernatant was removed and the beads were washed two times with 80% ethanol. After a final 5 min incubation with the caps open to allow the beads to dry, the DNA was eluted in 15 μL 10 mM TRIS buffer (pH 8.5).

The purified products were assembled into a pUC19 vector that was amplified by primers Puc-049cloning-R + Puc-049cloning-L (Table 2) using OneTaq polymerase (NEB). Assembly of the product into the pUC19 vector was by Gibson assembly [7, 8, 33], assembly reactions were electroporated into *E. coli* strain Epi300 (Epicentre), and resulting clones were selected on LB plates containing ampicillin at 100 μg mL^− 1^. Colonies were screened using 20 μL colony PCR reactions with primers pUC19-5’F and pUC19-3’R (OneTaq, NEB). Colonies containing a plasmid with an insert sequence of 339-bp were grown overnight in 5 mL LB broth and purified using the QIAprep miniprep kit (Qiagen). The insert sequences of the resulting plasmids were analyzed by Sanger DNA sequencing. For each treatment, 10-20 independent clones were sequenced using the pUC19-5’F primer.

### Data analysis

Samples recovered from Lanes 1 and 2, 3-8, which corresponded to assembly-only and EC treatments, respectively, were pooled and analyzed by DNA gel electrophoresis on a 2% agarose gel, and using the 1 Kb plus DNA ladder (Invitrogen) as a size standard. The indication of a successful experimental run was the presence of a 339-bp band. For the in-depth error analysis, the samples were cloned into pUC19 vectors and Sanger sequenced. Sequencing data was analyzed by aligning sequencing output files with the template DNA (Additional file 2). Each sequence alignment was inspected for errors in the newly assembled sequence. The errors were categorized in three groups: deletions, insertions, and substitutions. The sequences that had misincorporated oligonucleotides were treated as “mis-assemblies”. The error frequency per 1 kb (f) was calculated using Eq. 1 [34].

1 $$ \mathrm{f}=\frac{\sum \limits_{\mathrm{i}}^{\mathrm{n}}{\mathrm{x}}_{\mathrm{i}}\times 1000}{\mathrm{n}\times {\mathrm{l}}_{\mathrm{i}}} $$

where x_i_ is the number of errors in a single clone, n is the number of sequenced clones not including clones with mis-assemblies, and l_i_ is the length of a sequence in bases.

## Results

### Optimization of microfluidic PCR

**Fig. 6:**
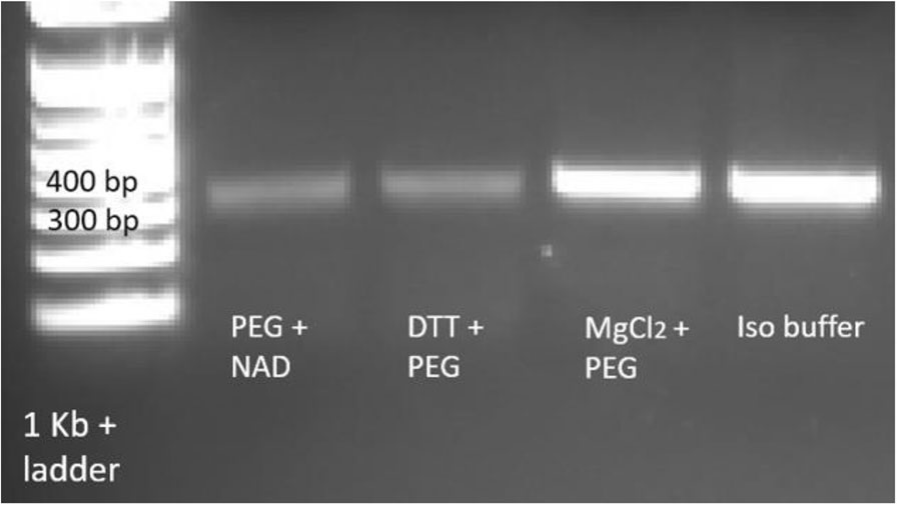
On-chip polymerase chain reaction performed with two components of the iso buffer as described in the text. DNA from the reactions was separated by agarose gel electrophoresis on a 2% agarose gel

Optimization of PCR on DMF demonstrated that the additives improved amplification efficiency. Control samples, which contained 0.1 U μL^− 1^ of Phusion polymerase, did not show any bands on the agarose gel (data not shown). On the other hand, PCR that contained the iso buffer used for DNA assembly resulted in the desired 339-bp bands. To determine the components of the iso buffer that contributed to the successful PCR reaction, we tested each component individually and in combination. When PEG 8000, DTT, NAD, and MgCl_2_ were added separately to the reaction, only PEG 8000 demonstrated some amplification of DNA template, but the result was not as good as PCR supplemented with the iso buffer (data not shown). Based on these results PEG 8000 was combined with either NAD, DTT, or MgCl_2_ to find out if DNA would be amplified at the same level as with the iso buffer. As seen in Fig. 6, the combination of 1.25 mM PEG 8000 and 1 mM MgCl_2_ showed comparable band intensity as the iso buffer. This result demonstrated that microfluidic PCR must be performed with an excess of Phusion enzyme and supplemented with additional MgCl_2_ and PEG 8000.

Multiple methods to reduce biofouling in microfluidics have been tested in this work. The only method that improved PCR yield and transport of droplets was the reduction of the electrowetting voltage from 300 V to 90 V during high-temperature PCR [18]. For more information refer to the Additional file 3: Video 2 included in Additional files.

### Optimization of microfluidic Gibson assembly

**Fig. 7:**
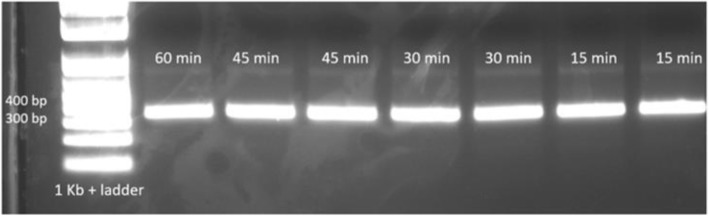
On-chip oligonucleotide assembly and PCR amplifications testing different assembly reaction durations. DNA from the reactions was separated by agarose gel electrophoresis on a 2% agarose gel

The first group of experiments tested optimum reaction time for oligonucleotide assembly. When we tested reaction times spanning 15-60 min, the bands for all tested times were of similar brightness (Fig. 7), suggesting the oligonucleotides were assembled in the 15-60 min time period.

**Fig. 8:**
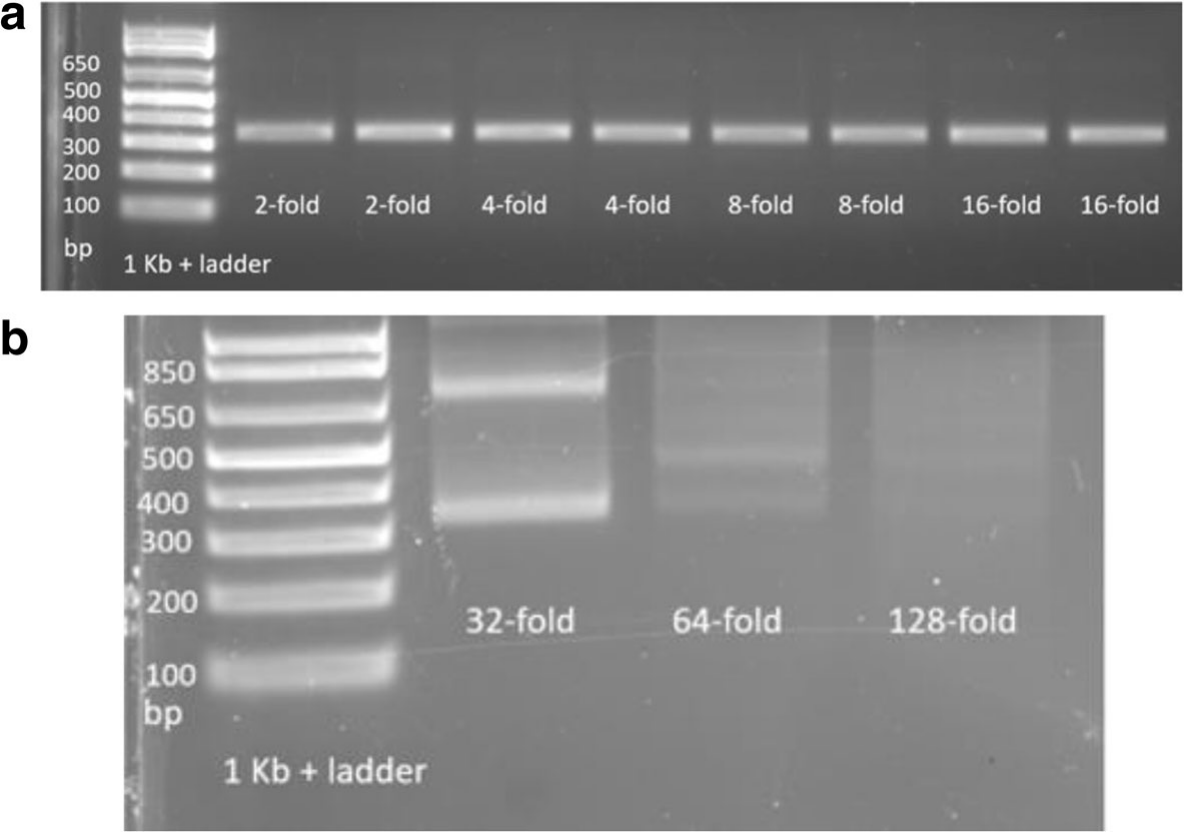
Dilution of the oligonucleotide assembly product (assembled with 50 nM each oligo) prior to PCR. **a** 2 to 16-fold and **b** 32 to 128-fold. After dilution and PCR, products were separated by agarose gel electrophoresis on 2% agarose gels

Figure 8 demonstrates the results of microfluidic assembly experiments with a dilution of assembly constructs prior to PCR. Dilution of the assembly constructs from 2-fold to 16-fold resulted in comparable amounts of the PCR product. However, 16-fold was the maximum dilution rate that could be achieved before the PCR template was too dilute to amplify. A dilution rate greater than 32-fold did not result in amplification of the assembly product.

**Fig. 9:**
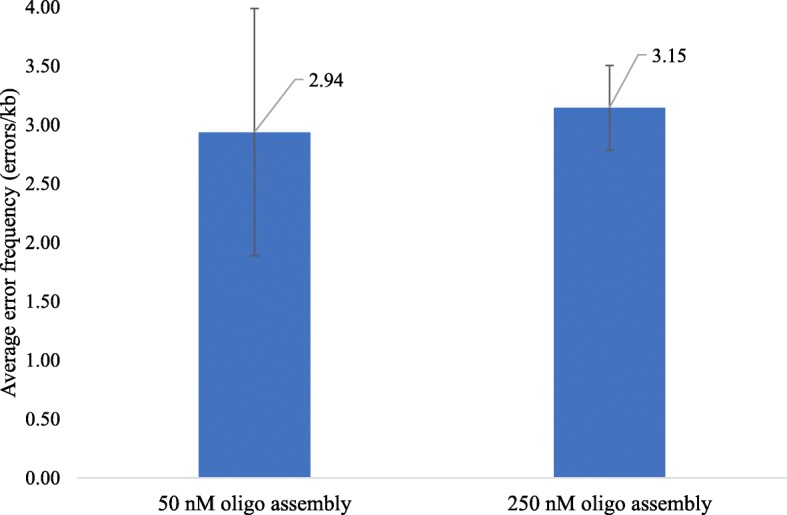
Average error frequency for sequences assembled from 250 nM and 50 nM oligonucleotides. Average error frequency from five independent experiments is plotted with error bars indicating one standard deviation from the mean

To investigate if the concentration of oligonucleotides in the assembly reaction could influence the fidelity of assembly constructs, two sets of samples obtained by the assembly of 50 nM or 250 nM oligonucleotides were sequenced. The average error rate from five separate runs for each oligonucleotides concentration is shown in Fig. 9. It was determined that the average error rate for 250 nM and 50 nM oligonucleotides was similar, at 3.15 errors kb^− 1^ and 2.94 errors kb^− 1^, respectively.

Single-base deletions comprised the bulk of errors. There was no preference for errors to occur between A/T or C/G bases. Both sets of samples had comparable percentages of deletions and the same amount of multiple-base deletions. The assembly of 50 nM oligonucleotides resulted in 80% deletion errors, whereas 250 nM assembly had 83% deletions, with the remainder being multiple base deletions, insertions or substitutions.

### Optimization of enzymatic error correction

**Fig. 10:**
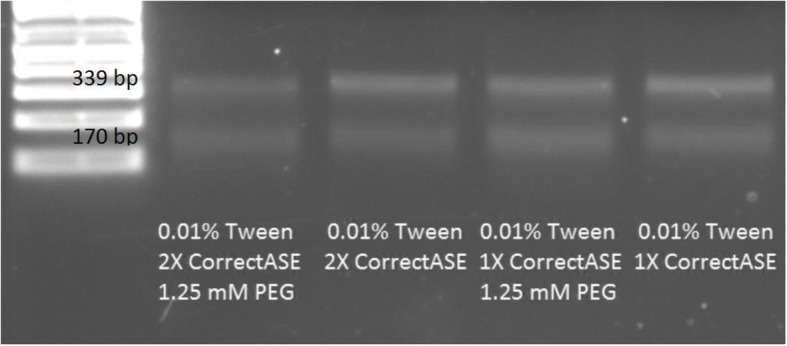
CorrectASE™ optimization on the DMF platform. DNA from the reactions was separated by agarose gel electrophoresis on a 2% agarose gel

An optimization of the error correction reaction was conducted to determine the reagents that reduce adsorption of CorrectASE™ on the oil/water interface and improve the performance of CorrectASE™ enzyme. The reactions were supplemented with either PEG 8000, Tween 20, and excess CorrectASE™ or combination of the additives. Our test for CorrectASE™ activity was to add a mixture of two PCR products with one containing a nucleotide mismatch in the sequence relative to the other at approximately the midpoint of the sequence. Thus, successful error correction led to cleavage of the full-length product and resulted in two bands (339-bp and 170-bp) on the agarose gel with comparable intensities. As seen in Fig. 10, the presence of 0.01% Tween 20, 2X CorrectASE™ and 1.25 mM of PEG 8000 in the reaction droplet gave the most even brightness in both bands on the agarose gel.

### Validation of DMF protocol for assembly with error correction

**Fig. 11:**
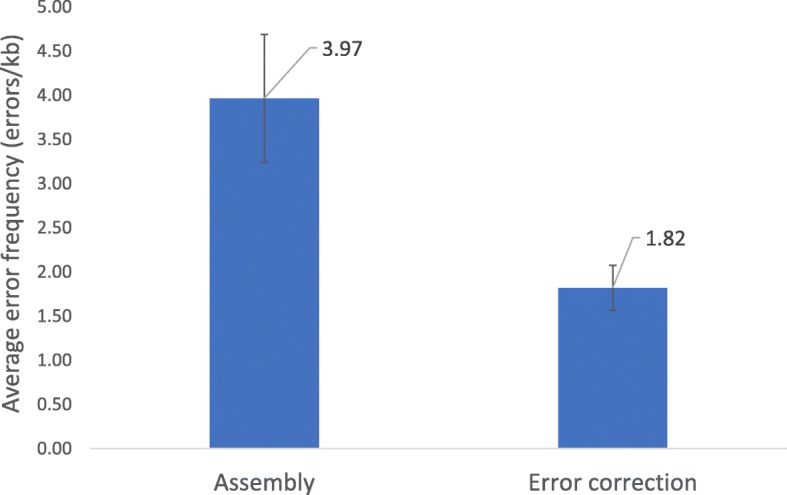
Average error frequency of assembly samples followed by CorrectASE™ treatment. The average error rate of three independent experiments is plotted with error bars indicating one standard deviation from the mean

The error analysis of DNA assembly of 12 oligonucleotides followed by CorrectASE™ treatment is shown in Fig. 11 and Additional file 2. The average error frequency of assembly samples from three separate runs was found to be about 4 errors kb^− 1^, which corresponds to what is widely reported for phosphoramidite DNA synthesis chemistry, where error rates of approximately 1:200 are typical. The average error frequency of the samples after error correction was about 2 errors kb^− 1^, which corresponds to an average error reduction by 2-fold. The average error reduction using a conventional benchtop protocol with the same sequence was found to be about 10-fold (data not shown).

As seen in Table 3, the enzyme was effective in removing deletion and insertion errors but failed to remove substitutions. Overall, error correction was successful, and in all three experiments about half of the sequenced clones were found to be error-free.

**Table 3:** Error analysis of DNA obtained using DMF protocol

	Run 1		Run 2		Run 3	
	Assembly	EC	Assembly	EC	Assembly	EC
Deletions	10	4	9	4	10	4
Insertions	0	0	2	0	1	0
Substitutions	3	3	1	0	0	1
Total clones sequenced	8	14	10	10	9	7
Total of clones with correct sequences	0	7	1	5	0	2

## Discussion

### Optimization of microfluidic PCR

The results of the microfluidic PCR experiments demonstrated that due to the high surface-to-volume ratio, reactions performed on the microfluidic device show a strong dependence on surface interactions. Protein molecules can adsorb at the oil/water interface, which reduces the surface tension over time [27, 35]. Additionally, adsorption of a protein at the droplet aqueous/oil interface could facilitate segregation of hydrophobic groups that may lead to change in protein conformation and inactivation. At high temperatures, the exposed hydrophobic groups of the protein could lead to protein denaturation. The combined effect of protein adsorption and denaturation could reduce the amount of available enzyme and decrease reaction efficiency. It has been reported previously that to achieve amplification efficiency similar to a benchtop PCR, the amount of polymerase must be increased up to 10-fold in microfluidic droplets [36, 37]. The results of PCR experiments presented here demonstrated that sufficient and repeatable PCR amplification could be achieved with a 5-fold increase of Phusion polymerase.

The efficiency and specificity of PCR are affected by the Mg^2+^ concentration. Magnesium ions help the polymerase to fold in the active conformation [38]. Also, Mg^2+^ stabilizes dsDNA and increases the melting temperature (T_m_) of primers. Thus, it is crucial to have the correct amount of free magnesium, and this concentration must often be optimized for each primer pair. It has been observed that the concentration of free Mg^2+^ can be decreased because of precipitation on microfluidic surfaces, capture by chelating agents present in reagents and storage buffers, and by binding to dNTPs [37]. According to the Phusion polymerase product literature (Thermo), the optimum concentration of MgCl_2_ is between 0.5-1 mM. The experimental results demonstrated that the addition of 0.5-1 mM of magnesium to the 1.5 mM MgCl_2_ present in Phusion HF buffer, improved polymerase activity, but this effect was inconsistent from lane to lane. However, it was shown that the synergistic effect of magnesium and PEG 8000 created favorable conditions for PCR amplification (Fig. 6).

Polyethylene glycol (PEG) is recognized as a molecular crowding agent and frequently used as a PCR enhancer and an enzyme immobilization agent [39, 40]. Molecular crowding creates the conditions similar to a natural cell environment in which the enzyme was evolved. It was reported that macromolecular crowding affects the enzyme reaction kinetics by increasing the viscosity of a medium that in turn influences the diffusion of reagents. Also, the polymers preserve native protein conformation and facilitate binding to a substrate. It has been shown that PEG 8000 stabilized Taq polymerase at high temperatures [39, 41]. Since Phusion is a polymerase, it is possible that PEG 8000 formed weak bonds with the enzyme and reduced hydrophobic interactions with the Teflon coating. As a result, the activity of the enzyme was increased and amplification yield was improved [42]. Consequently, microfluidic PCR is affected by adsorption as well as by interactions of reaction components with interfaces. In order to achieve amplification on the DMF, the reaction must be carried out with the final concentration of 0.1 U μL^− 1^ of Phusion (a 5-fold increase relative to standard benchtop conditions), 0.5-1 mM of MgCl_2_, and 0.625-1.25 mM of PEG 8000.

Improved PCR yield at 90 V showed that at the lower voltage, the oil film between the aqueous droplet and the Teflon coated surface stayed intact and eliminated hydrophobic interactions between the polymerase and the surface. According to Kleinert et al., the actuation voltage has a significant influence on the oil film [31]. At high actuation voltage when the droplet moves, the film becomes unstable, breaks down, and tiny oil droplets get trapped under the aqueous phase.

In addition, excess surfactant destabilizes the oil film. Mohajeri and colleagues demonstrated that the critical micellar concentration of nonionic surfactants such as Tween 20 decreases at higher temperatures [32]. Thus, in the denaturation zone, less surfactant is necessary to reduce the surface tension. If there is an excessive amount of surfactant, the oil film becomes unstable, and the adsorption of the protein occurs, which is further enhanced at high temperatures. It is important to use lower voltage and minimize the amount of Tween 20 to avoid loss of Phusion polymerase and subsequent droplet transport failure.

### Optimization of microfluidic Gibson assembly

Microfluidic DNA assembly protocols developed in this work produce results similar to the results published in the literature. Our results show that even 15 min was an acceptable length of time for an efficient microfluidic DNA assembly. In bench-top reactions, DNA assembly reactions proceed in reaction times between 15 and 60 min [26, 33, 43].

Dilution of the assembly product prior to the PCR amplification is an additional step that should be included in a microfluidic Gibson assembly protocol. Since the goal was to assemble the product that had the minimum number of errors, it was important to remove unreacted oligonucleotides, oligonucleotide fragments, and mis-assemblies that were present at a low level before amplification. Based on these results, we kept the dilution of the assembly product no greater than 16-fold. If the dilution step before PCR is employed, the amplification mix must contain 0.1 U μL^− 1^ of Phusion, 0.625 mM PEG 8000, and 0.5 mM MgCl_2_.

The results of the error analysis suggest that the concentration of oligonucleotides during assembly did not affect the fidelity of the resulting sequence. Both DNA assembly methods demonstrated an error frequency in the 1-10 errors kb^− 1^ range, which was similar to the values reported in the literature for microfluidic DNA assembly [9]. For instance, Saem et al. reported 1.9 errors kb^− 1^, Sequeira et al. reported 3.45 errors kb^− 1^, Kosuri et al. reported 4 errors kb^− 1^, and Yehezkel et al. reported 2.2 errors kb^− 1^ [18, 44–46]. The analysis of error types demonstrated that the majority of errors belonged to single-base deletions with a small percentage of insertions and substitutions. These results are comparable to 75.6% deletions, 2.2% insertions, and 22.2% substitutions, obtained by Sequeira et al. [46]*.* However, several clones in both data sets had mis-incorporated oligonucleotides. This issue could be solved by improving the design of the overlapping oligonucleotide sequences. Since the 50 nM oligonucleotide dataset had 1.5 times more clones with mis-assemblies, degradation of some oligonucleotides by T5 exonuclease could be the cause of mis-incorporation. The results demonstrated that the Gibson assembly method performed on the DMF device is efficient. The error frequencies for microfluidic synthesized sequences are in line with those found for benchtop DNA synthesis in the published literature.

### Optimization of enzymatic error correction

Since the best CorrectASE™ activity was obtained with 0.01% Tween, 2X CorrectASE™, and 1.25 mM PEG additives, the adsorption of the enzyme on the oil/water interface of the aqueous droplet is the most likely explanation of error correction in previous runs. According to Baldursdottir et al., protein molecules tend to aggregate on the oil/water interface in a multilayer. The adsorption rate is affected by the molecular weight and a saturation concentration. Large protein molecules tend to adsorb faster than small ones due to the large surface area available for contact with the interface. Also, hydrophobic proteins tend to adsorb more due to interactions with the hydrophobic coated surface [47]. If some of the protein molecules adsorb on the interface, hydrophobic and hydrophilic groups will rearrange, and it will cause the protein to change conformation, leading to a loss of activity, and the reaction will not proceed with the maximum yield.

We previously showed for PCR reactions that the presence of a molecular crowding agent such as PEG significantly increased the activity of Phusion polymerase. According to Sasaki et al., the activity of DNase I to degrade supercoiled DNA and linear DNA was improved in the presence of 20% *w*/*v* PEG [40]. A kinetic analysis demonstrated that the rate of the DNA cleavage reaction increased with the increasing of concentration of PEG. However, molecular crowding did not improve the activity of Exonuclease III and inhibited the activity of Exonuclease I [40]. Consequently, macromolecular crowding could be the reason why the error correction reaction was improved on the DMF platform with the addition of PEG.

Surfactants in digital microfluidic electrowetting on dielectric (EWOD) are very important. The excess of surfactant can lead to a destruction of the oil film under the droplet, which could cause the adsorption of hydrophobic molecules on the microfluidic surface. Insufficient surfactant could also cause interface instability that in turn could cause the adsorption of enzymes on the oil/water interface. Usually, to be able to generate and manipulate the droplets on DMF the concentration of Tween 20 has to be 0.01-0.05% [18, 31]. However, enzymatic reactions contain multiple components which can potentially affect surface tension. Thus, the amount of Tween 20 has to be optimized for individual reactions. It has been demonstrated in this study that even the presence of 0.001% of Tween 20 in reaction droplets in conjunction with the excess of CorrectASE™ and PEG 8000 gives reproducible error correction results.

### Validation of DMF protocol for assembly with error correction

The results of our microfluidic protocol demonstrated that some inhibition of CorrectASE™ was still occurring during error correction on the DMF platform. Lower error reduction could also be related to over dilution of error correction products prior to PCR or errors in amplification. This suggests that further optimization is possible on the DMF device.

## Conclusion

Oligonucleotide assembly and error correction protocols for the Mondrian™ digital microfluidic device were developed. The process involved automation of the polymerase chain reaction, Gibson assembly of 12 oligonucleotides, and enzymatic error correction reaction with CorrectASE™. The final protocol consisted of the assembly of oligonucleotides, two PCR steps, and an error correction reaction. To achieve PCR amplification on the DMF platform, the reactions were supplemented with PEG, MgCl_2_, and 5-fold increased amount of polymerase (relative to benchtop conditions). The error correction reaction was supplemented with PEG, Tween 20, and an excess of CorrectASE™ (2-fold increase relative to benchtop conditions). The final protocol assembled DNA sequences with an average of 4 errors kb^− 1^ and reduced errors after error correction by 2-fold.

## Additional files

Additional file 1: Video demonstration of droplet liquid handling operations used in the study. (MP4 9178 kb)

Additional file 2: Sequencing reads aligned to a template. (PDF 1973 kb)

Additional file 3: Video demonstration of droplet movement during 300 V and 90 V PCR cycle. (MP4 19348 kb)

## Abbreviations

DMF: Digital microfluidics
DTT: Dithiothreitol
EC: Error correction
NAD: Nicotinamide adenine dinucleotide
PCR: Polymerase chain reaction
PEG: Polyethylene glycol
