# Supplementary material for: DNA assembly with error correction on a droplet digital microfluidics platform
Source: BMC Biotechnol. 2018 Jun 1;18:37. doi: 10.1186/s12896-018-0439-9 (PMC5984785; doi:10.1186/s12896-018-0439-9)
Supplement: Supplementary file 2 — Sequencing reads aligned to a template. (PDF 1973 kb) [file 12896_2018_439_MOESM2_ESM.pdf]

Additional File 3

This file contains the Sanger DNA sequencing reads aligned to the reference sequence containing the assembled influenza virus HA fragment. Each sequence was obtained from an independently cloned DNA fragment and the aligned sequence extends onto two pages; the first page of the alignment begins on the page number shown below. The influenza virus sequence assembled from oligonucleotides is annotated as “AVIAN_H9N2_HA_2C00049”. The vector sequences flanking the assembled sequence are annotated as “hook 1” and “hook 2”.

Sample - Treatment	Page number
Run 1-Assembly-only	2
Run 1-Error-correction	6
Run 2-Assembly-only	12
Run 2-Error-correction	18
Run 3-Assembly-only	24
Run 3-Error-correction	28

Template Alignment: Run 1 Assembly Only

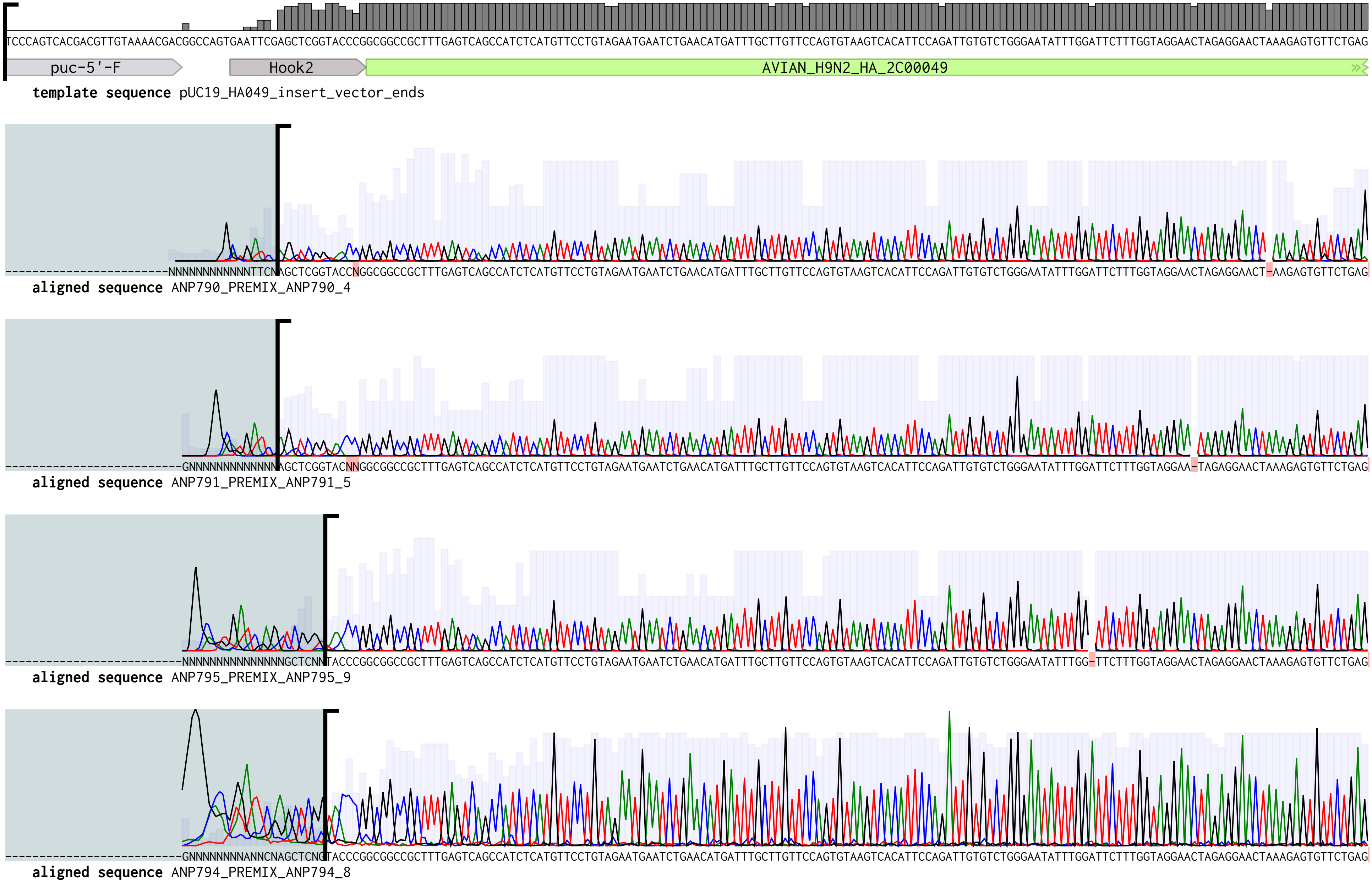

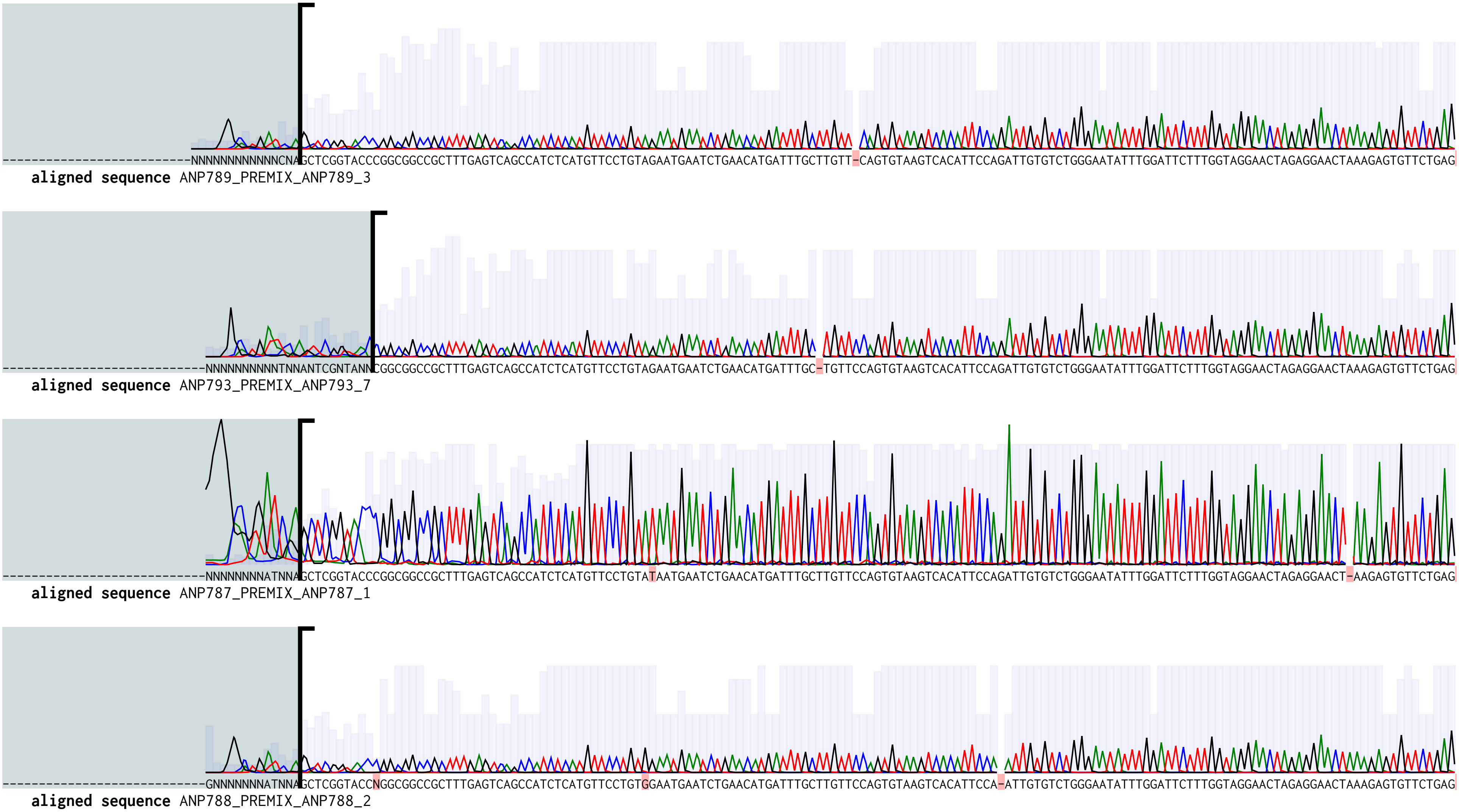

-NNNNNNNNNNNNNCNAGCTCGGTACCCGGCGGCCGCTTTGAGTCAGCCATCTCATGTTCCTGTAGAATGAATCTGAACATGATTTGCTTGTT-CAGTGTAAGTCACATTCCAGATTGTGTCTGGGAATATTTGGATTCTTTGGTAGGAACTAGAGGAACTAAAGAGTGTTCTGAG
aligned sequence ANP789_PREMIX_ANP789_3
-NNNNNNNNNNTNNANTCGNTANNCGGCGGCCGCTTTGAGTCAGCCATCTCATGTTCCTGTAGAATGAATCTGAACATGATTTGC-TGTTCCAGTGTAAGTCACATTCCAGATTGTGTCTGGGAATATTTGGATTCTTTGGTAGGAACTAGAGGAACTAAAGAGTGTTCTGAG
aligned sequence ANP793_PREMIX_ANP793_7
-NNNNNNNNATNNAGCTCGGTACCCGGCGGCCGCTTTGAGTCAGCCATCTCATGTTCCTGTATAATGAATCTGAACATGATTTGCTTGTTCCAGTGTAAGTCACATTCCAGATTGTGTCTGGGAATATTTGGATTCTTTGGTAGGAACTAGAGGAACT-AAGAGTGTTCTGAG
aligned sequence ANP787_PREMIX_ANP787_1
-GNNNNNNNATNNAGCTCGGTACCNGGCGGCCGCTTTGAGTCAGCCATCTCATGTTCCTGTGGAATGAATCTGAACATGATTTGCTTGTTCCAGTGTAAGTCACATTCCA-ATTGTGTCTGGGAATATTTGGATTCTTTGGTAGGAACTAGAGGAACTAAAGAGTGTTCTGAG
aligned sequence ANP788_PREMIX_ANP788_2

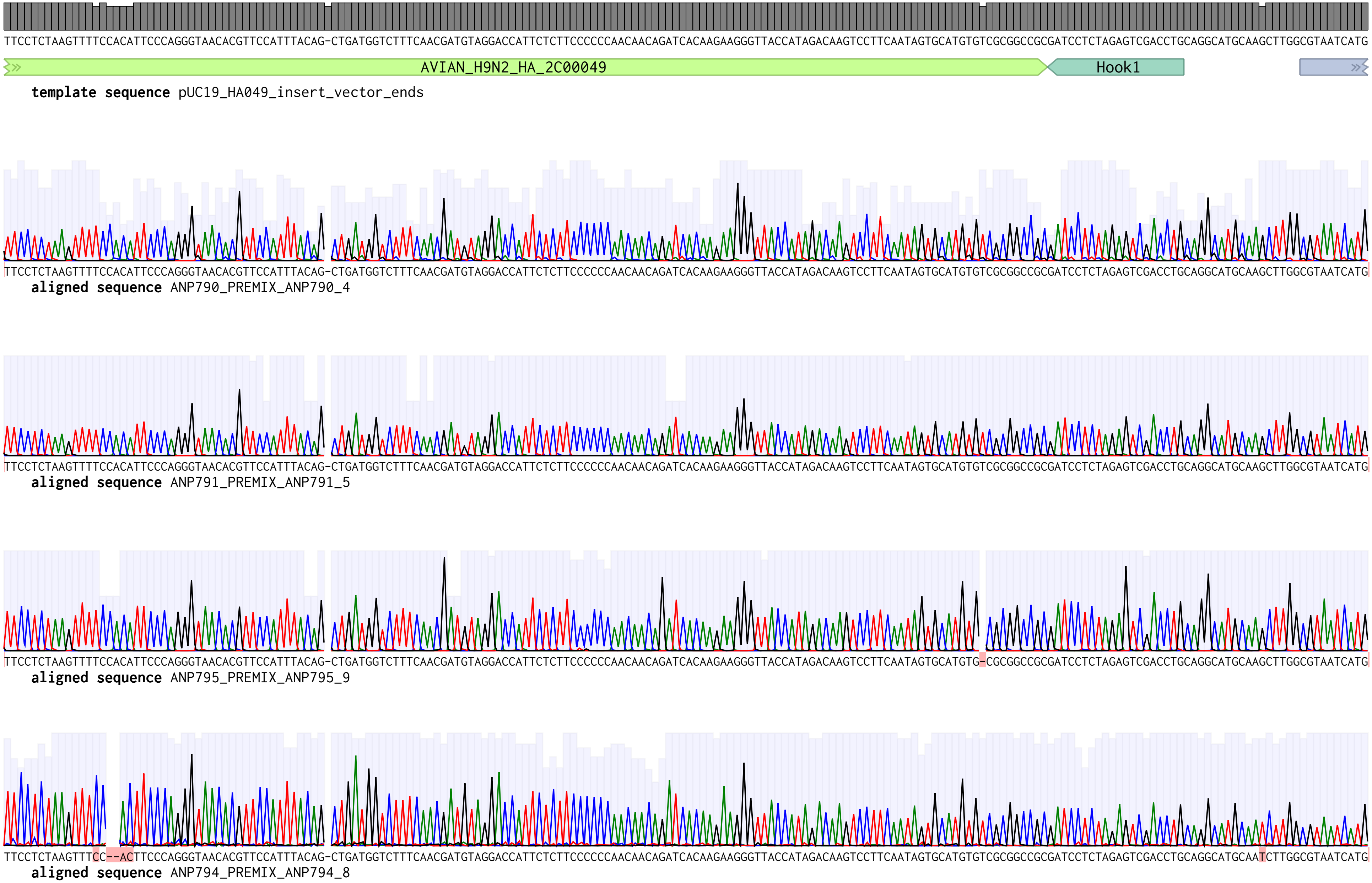

TTCCTCTAAGTTTTCCACATTCCCAGGGTAACACGTTCCATTTACAG-CTGATGGTCTTTCAACGATGTAGGACCATTCTCTTCCCCCAACAACAGATCACAAGAAGGGTTACCATAGACAAGTCCTTCAATAGTGCATGTGTCGCGGCCGCGATCCTCTAGAGTCGACCTGCAGGCATGCAAGCTTGGCGTAATCATG
AVIAN_H9N2_HA_2C00049
Hook1
template sequence pUC19_HA049_insert_vector_ends
TTCCTCTAAGTTTTCCACATTCCCAGGGTAACACGTTCCATTTACAG-CTGATGGTCTTTCAACGATGTAGGACCATTCTCTTCCCCCAACAACAGATCACAAGAAGGGTTACCATAGACAAGTCCTTCAATAGTGCATGTGTCGCGGCCGCGATCCTCTAGAGTCGACCTGCAGGCATGCAAGCTTGGCGTAATCATG
aligned sequence ANP790_PREMIX_ANP790_4
TTCCTCTAAGTTTTCCACATTCCCAGGGTAACACGTTCCATTTACAG-CTGATGGTCTTTCAACGATGTAGGACCATTCTCTTCCCCCAACAACAGATCACAAGAAGGGTTACCATAGACAAGTCCTTCAATAGTGCATGTGTCGCGGCCGCGATCCTCTAGAGTCGACCTGCAGGCATGCAAGCTTGGCGTAATCATG
aligned sequence ANP791_PREMIX_ANP791_5
TTCCTCTAAGTTTTCCACATTCCCAGGGTAACACGTTCCATTTACAG-CTGATGGTCTTTCAACGATGTAGGACCATTCTCTTCCCCCAACAACAGATCACAAGAAGGGTTACCATAGACAAGTCCTTCAATAGTGCATGTG-CGCGGCCGCGATCCTCTAGAGTCGACCTGCAGGCATGCAAGCTTGGCGTAATCATG
aligned sequence ANP795_PREMIX_ANP795_9
TTCCTCTAAGTTTCC--ACTTCCCAGGGTAACACGTTCCATTTACAG-CTGATGGTCTTTCAACGATGTAGGACCATTCTCTTCCCCCAACAACAGATCACAAGAAGGGTTACCATAGACAAGTCCTTCAATAGTGCATGTGTCGCGGCCGCGATCCTCTAGAGTCGACCTGCAGGCATGCAATCTTGGCGTAATCATG
aligned sequence ANP794_PREMIX_ANP794_8

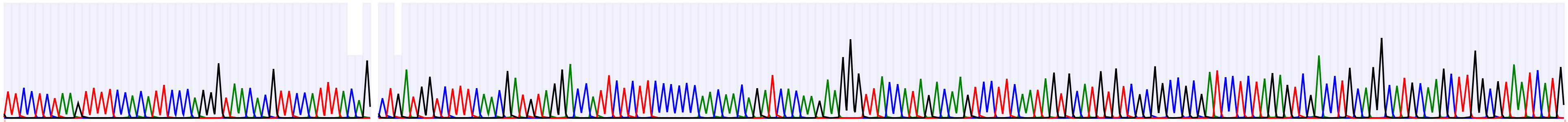

TTCCTCTAAGTTTTCCACATTCCCAGGGTAACACGTTCCATTTACAG-CTGATGGTCTTTCAACGATGTAGGACCATTCTCTTCCCCCCAACAACAGATCACAAGAAGGGTTACCATAGACAAGTCCTTCAATAGTGCATGTGTCGCGGCCGCGATCCTCTAGAGTCGACCTGCAGGCATGCAAGCTTGGCGTAATCATG

aligned sequence ANP789_PREMIX_ANP789_3

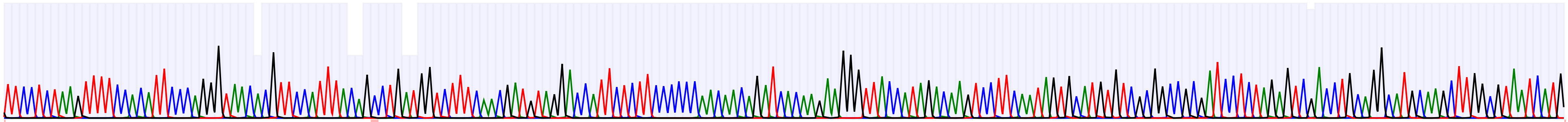

TTCCTCTAAGTTTTCCACATTCCCAGGGTAACACGTTCCATTTACAGCCTGATGGTCTTTCAACGATGTAGGACCATTCTCTTCCCCCCAACAACAGATCACAAGAAGGGTTACCATAGACAAGTCCTTCAATAGTGCATGTGTCGCGGCCGCGATCCTCTAGAGTCGACCTGCAGGCATGCAAGCTTGGCGTAATCATG

aligned sequence ANP793_PREMIX_ANP793_7

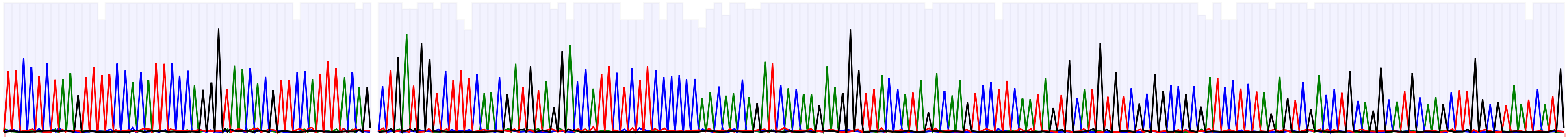

TTCCTCTAAGTTTTCCACATTCCCAGGGTAACACGTTCCATTTACAG-CTGATGGTCTTTCAACGATGTAGGACCATTCTCTTCCCCCCAACAACAGATCACAAGAAGGGTTACCATAGACAAGTCCTTCAATAGTGCATGTGTCGCGGCCGCGATCCTCTAGAGTCGACCTGCAGGCATGCAAGCTTGGCGTAATCATG

aligned sequence ANP787_PREMIX_ANP787_1

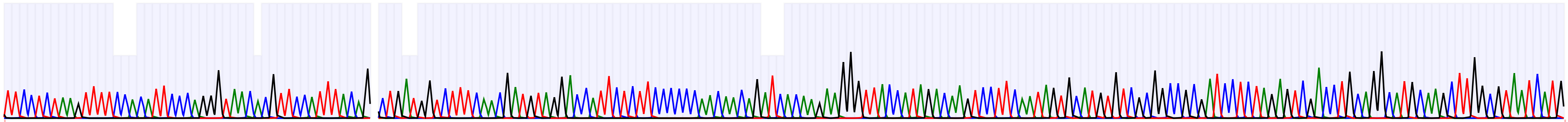

TTCCTCTAAGTTTTCCACATTCCCAGGGTAACACGTTCCATTTACAG-CTGATGGTCTTTCAACGATGTAGGACCATTCTCTTCCCCCCAACAACAGATCACAAGAAGGGTTACCATAGACAAGTCCTTCAATAGTGCATGTGTCGCGGCCGCGATCCTCTAGAGTCGACCTGCAGGCATGCAAGCTTGGCGTAATCATG

aligned sequence ANP788_PREMIX_ANP788_2

Template Alignment: Run 1 Error Correction

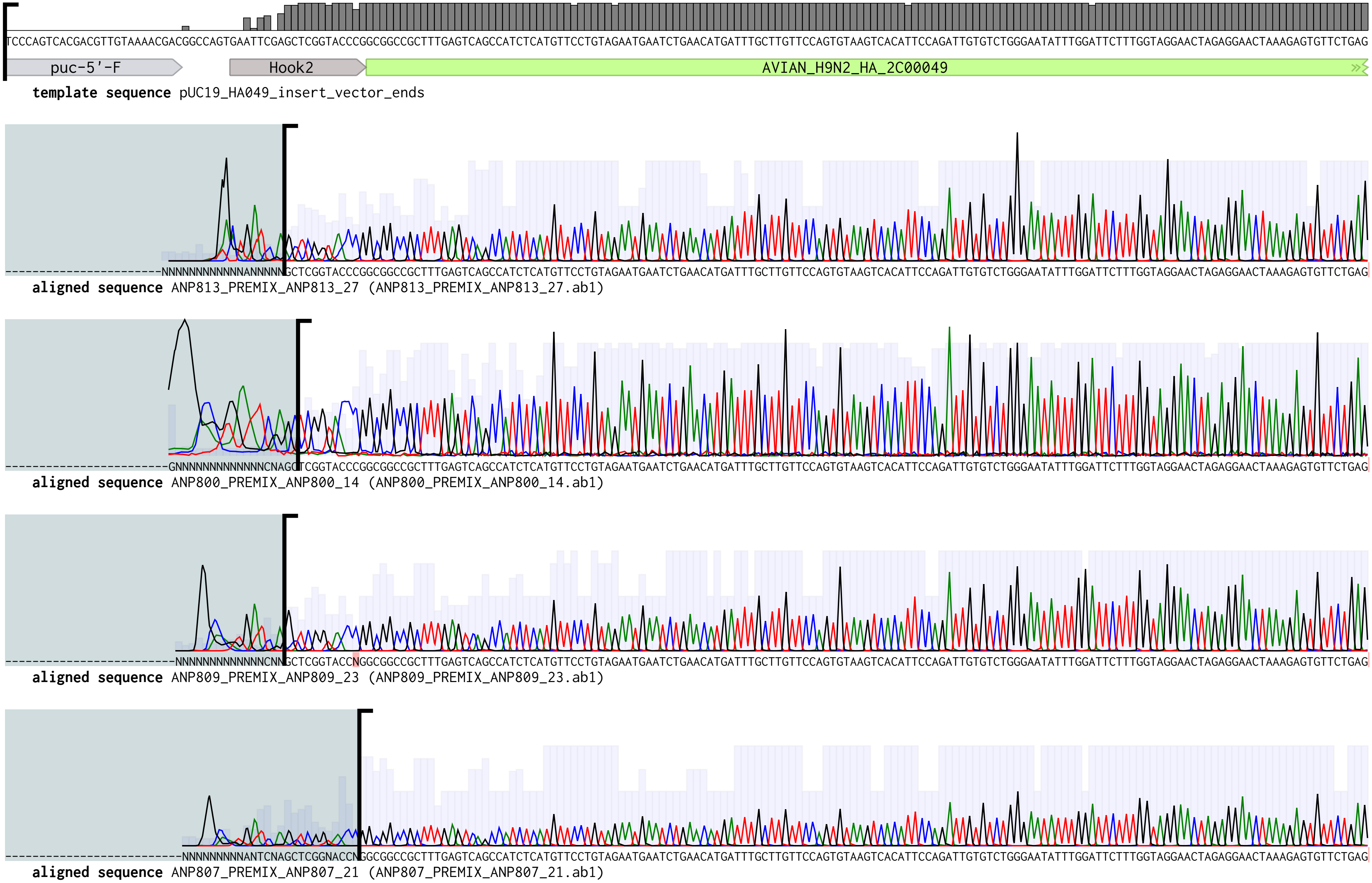

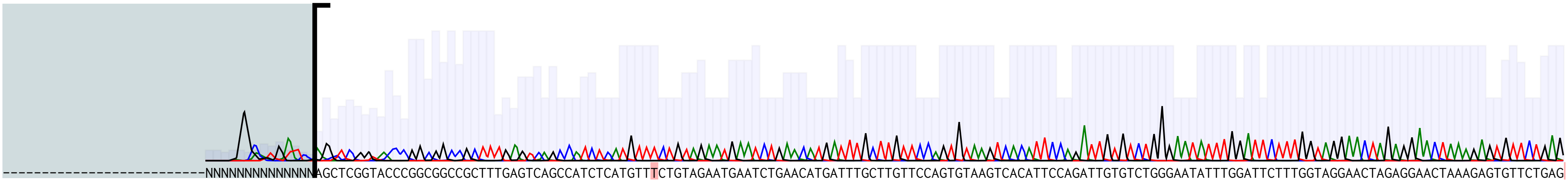

aligned sequence ANP808_PREMIX_ANP808_22 (ANP808_PREMIX_ANP808_22.ab1)

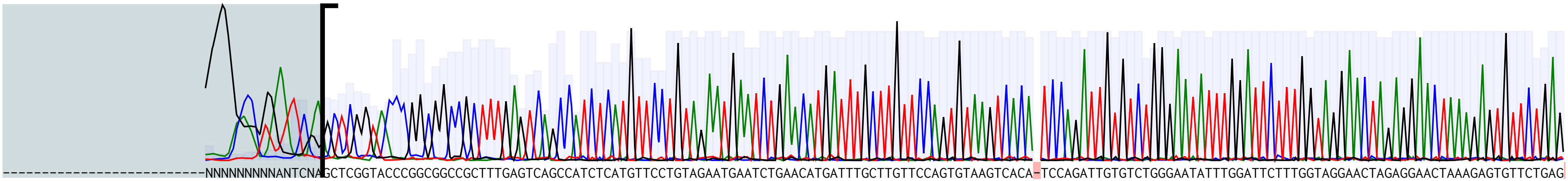

aligned sequence ANP802_PREMIX_ANP802_16 (ANP802_PREMIX_ANP802_16.ab1)

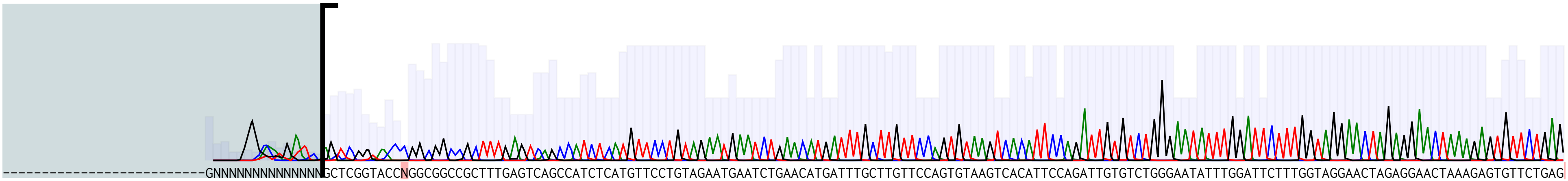

aligned sequence ANP806_PREMIX_ANP806_20 (ANP806_PREMIX_ANP806_20.ab1)

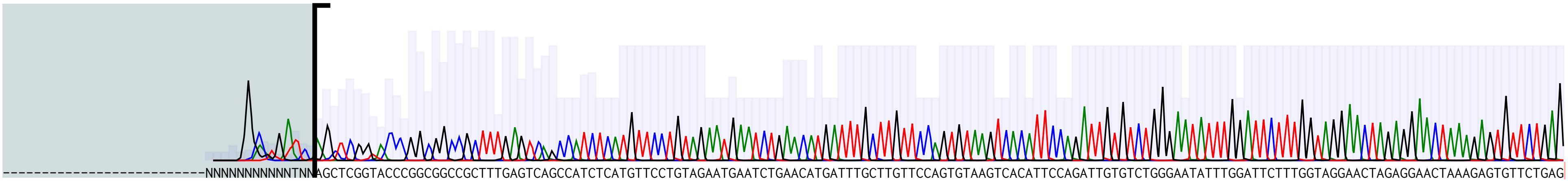

aligned sequence ANP798_PREMIX_ANP798_12 (ANP798_PREMIX_ANP798_12.ab1)

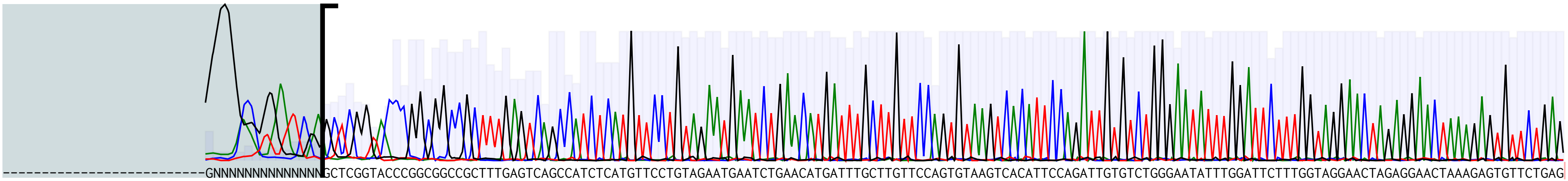

aligned sequence ANP799_PREMIX_ANP799_13 (ANP799_PREMIX_ANP799_13.ab1)

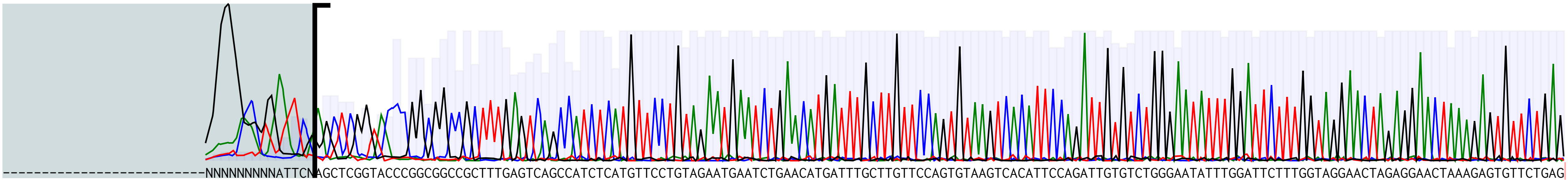

aligned sequence ANP811_PREMIX_ANP811_25 (ANP811_PREMIX_ANP811_25.ab1)

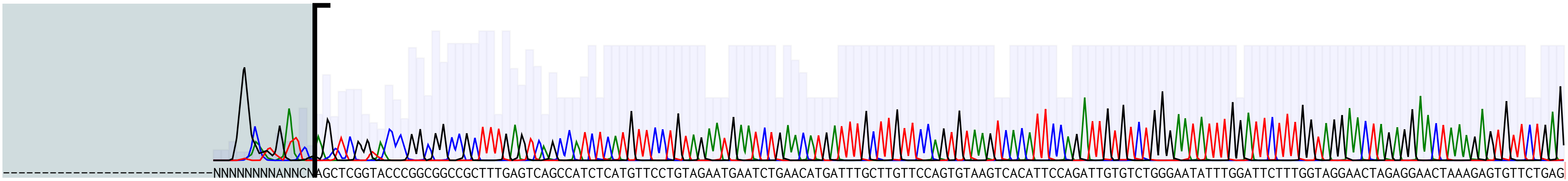

aligned sequence ANP814_PREMIX_ANP814_28 (ANP814_PREMIX_ANP814_28.ab1)

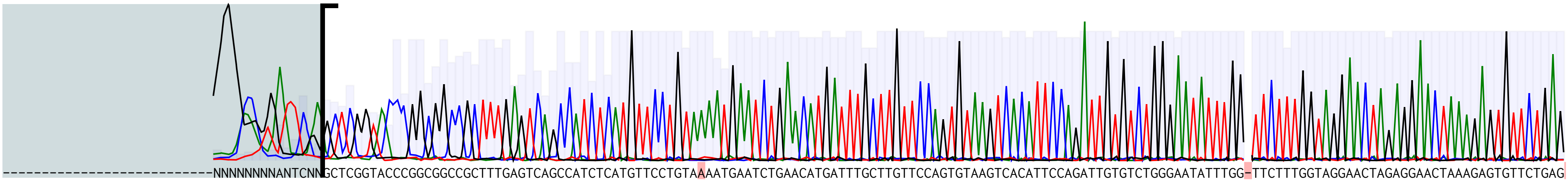

aligned sequence ANP804_PREMIX_ANP804_18 (ANP804_PREMIX_ANP804_18.ab1)

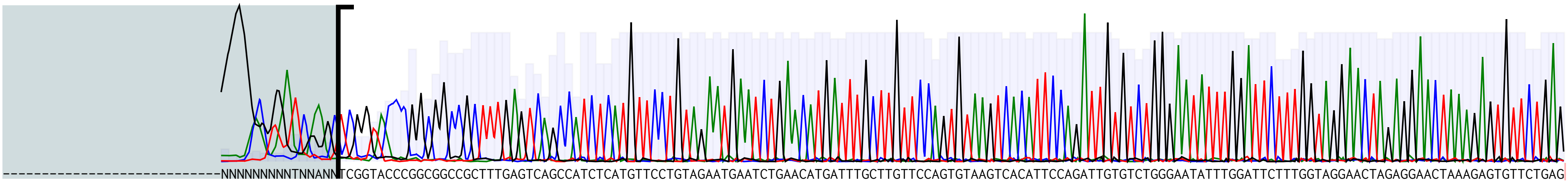

aligned sequence ANP812_PREMIX_ANP812_26 (ANP812_PREMIX_ANP812_26.ab1)

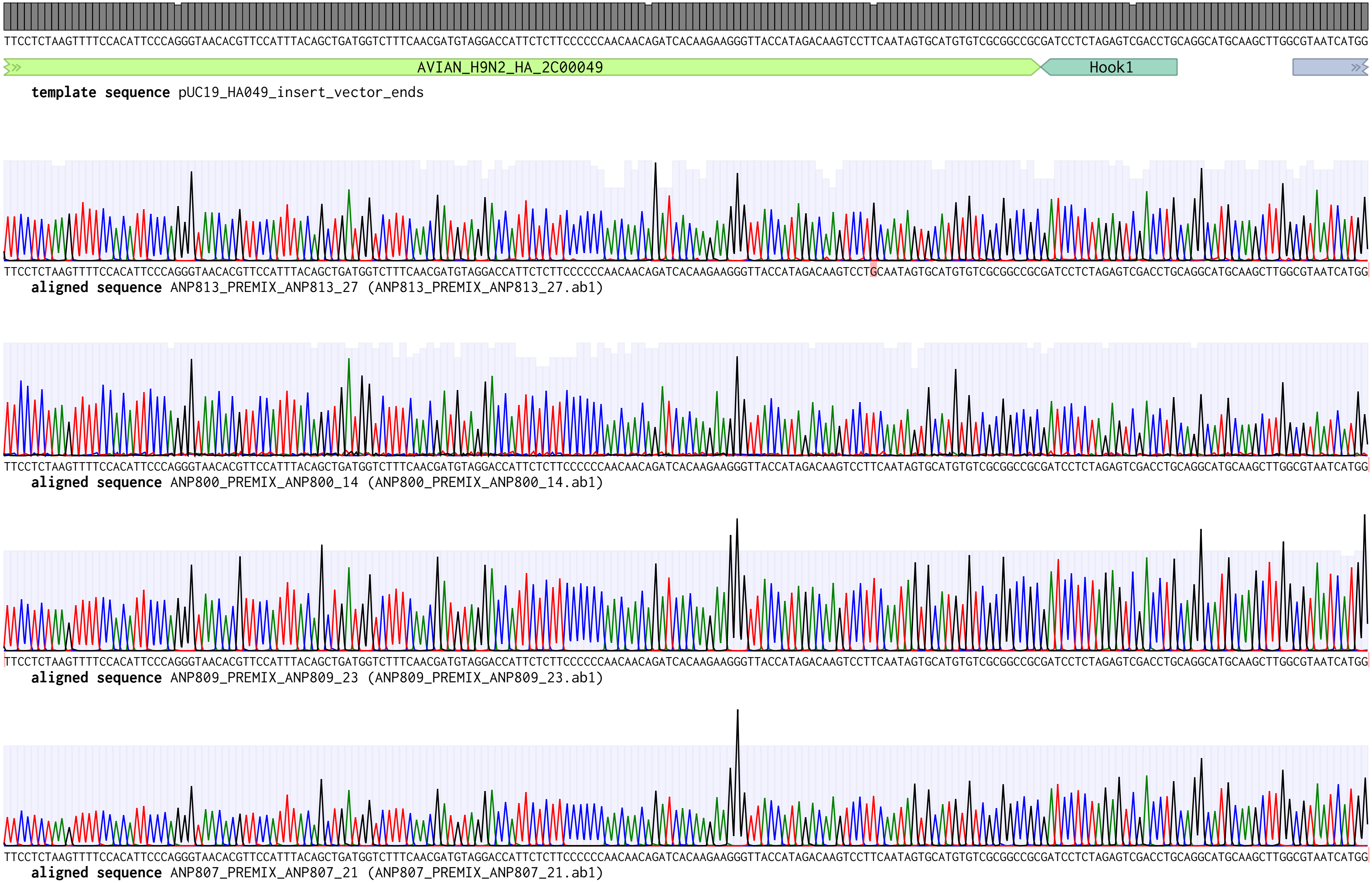
TTCCTCTAAGTTTTCCACATTCCCAGGGTAACACGTTCCATTTACAGCTGATGGTCTTTCAACGATGTAGGACCATTCTCTTCCCCCCAACAACAGATCACAAGAAGGGTTACCATAGACAAGTCCTTCAATAGTGCATGTGTCGCGGCCGCGATCCTCTAGAGTCGACCTGCAGGCATGCAAGCTTGGCGTAATCATGG
AVIAN_H9N2_HA_2C00049
Hook1
template sequence pUC19_HA049_insert_vector_ends
TTCCTCTAAGTTTTCCACATTCCCAGGGTAACACGTTCCATTTACAGCTGATGGTCTTTCAACGATGTAGGACCATTCTCTTCCCCCCAACAACAGATCACAAGAAGGGTTACCATAGACAAGTCCTGCAATAGTGCATGTGTCGCGGCCGCGATCCTCTAGAGTCGACCTGCAGGCATGCAAGCTTGGCGTAATCATGG
aligned sequence ANP813_PREMIX_ANP813_27 (ANP813_PREMIX_ANP813_27.ab1)
TTCCTCTAAGTTTTCCACATTCCCAGGGTAACACGTTCCATTTACAGCTGATGGTCTTTCAACGATGTAGGACCATTCTCTTCCCCCCAACAACAGATCACAAGAAGGGTTACCATAGACAAGTCCTTCAATAGTGCATGTGTCGCGGCCGCGATCCTCTAGAGTCGACCTGCAGGCATGCAAGCTTGGCGTAATCATGG
aligned sequence ANP800_PREMIX_ANP800_14 (ANP800_PREMIX_ANP800_14.ab1)
TTCCTCTAAGTTTTCCACATTCCCAGGGTAACACGTTCCATTTACAGCTGATGGTCTTTCAACGATGTAGGACCATTCTCTTCCCCCCAACAACAGATCACAAGAAGGGTTACCATAGACAAGTCCTTCAATAGTGCATGTGTCGCGGCCGCGATCCTCTAGAGTCGACCTGCAGGCATGCAAGCTTGGCGTAATCATGG
aligned sequence ANP809_PREMIX_ANP809_23 (ANP809_PREMIX_ANP809_23.ab1)
TTCCTCTAAGTTTTCCACATTCCCAGGGTAACACGTTCCATTTACAGCTGATGGTCTTTCAACGATGTAGGACCATTCTCTTCCCCCCAACAACAGATCACAAGAAGGGTTACCATAGACAAGTCCTTCAATAGTGCATGTGTCGCGGCCGCGATCCTCTAGAGTCGACCTGCAGGCATGCAAGCTTGGCGTAATCATGG
aligned sequence ANP807_PREMIX_ANP807_21 (ANP807_PREMIX_ANP807_21.ab1)

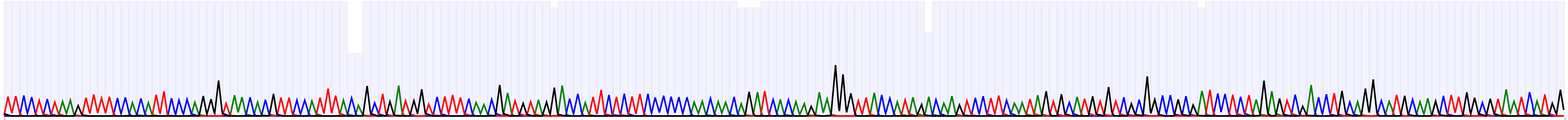

TTCCTCTAAGTTTTCCACATTCCCAGGGTAACACGTTCCATTTACAGCTGATGGTCTTTCAACGATGTAGGACCATTCTCTTCCCCCCAACAACAGATCACAAGAAGGGTTACCATAGACAAGTCCTTCAATAGTGCATGTGTCGCGGCCGCGATCCTCTAGAGTCGACCTGCAGGCATGCAAGCTTGGCGTAATCATGG

aligned sequence ANP808_PREMIX_ANP808_22 (ANP808_PREMIX_ANP808_22.ab1)

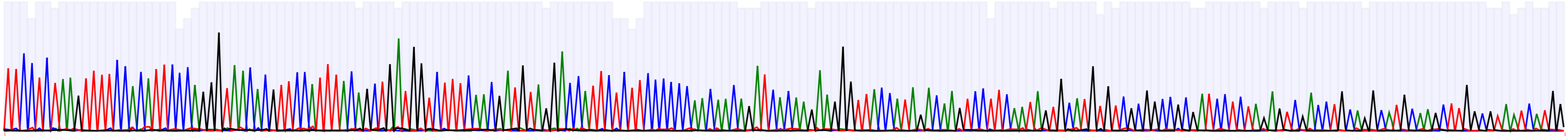

TTCCTCTAAGTTTTCCACATTCCCAGGGTAACACGTTCCATTTACAGCTGATGGTCTTTCAACGATGTAGGACCATTCTCTTCCCCCCAACAACAGATCACAAGAAGGGTTACCATAGACAAGTCCTTCAATAGTGCATGTGTCGCGGCCGCGATCCTCTAGAGTCGACCTGCAGGCATGCAAGCTTGGCGTAATCATGG

aligned sequence ANP802_PREMIX_ANP802_16 (ANP802_PREMIX_ANP802_16.ab1)

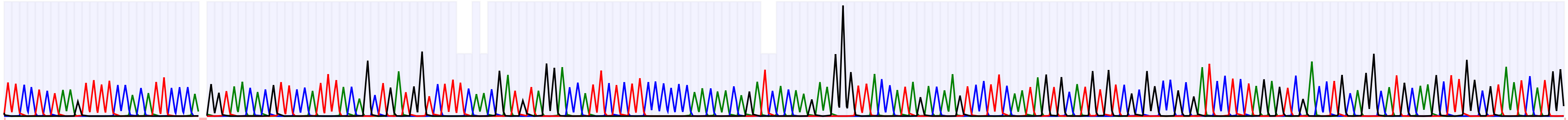

TTCCTCTAAGTTTTCCACATTCCCA-GGTAACACGTTCCATTTACAGCTGATGGTCTTTCAACGATGTAGGACCATTCTCTTCCCCCCAACAACAGATCACAAGAAGGGTTACCATAGACAAGTCCTTCAATAGTGCATGTGTCGCGGCCGCGATCCTCTAGAGTCGACCTGCAGGCATGCAAGCTTGGCGTAATCATGG

aligned sequence ANP806_PREMIX_ANP806_20 (ANP806_PREMIX_ANP806_20.ab1)

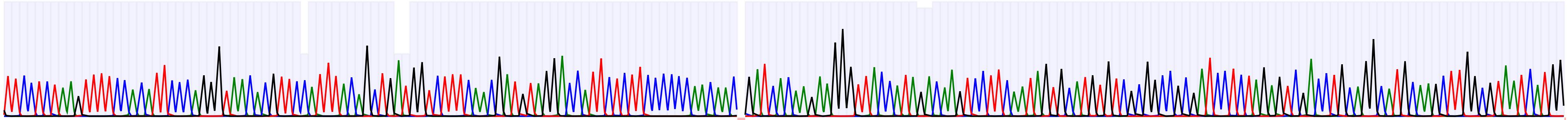

TTCCTCTAAGTTTTCCACATTCCCAGGGTAACACGTTCCATTTACAGCTGATGGTCTTTCAACGATGTAGGACCATTCTCTTCCCCCCAACAAC-GATCACAAGAAGGGTTACCATAGACAAGTCCTTCAATAGTGCATGTGTCGCGGCCGCGATCCTCTAGAGTCGACCTGCAGGCATGCAAGCTTGGCGTAATCATGG

aligned sequence ANP798_PREMIX_ANP798_12 (ANP798_PREMIX_ANP798_12.ab1)

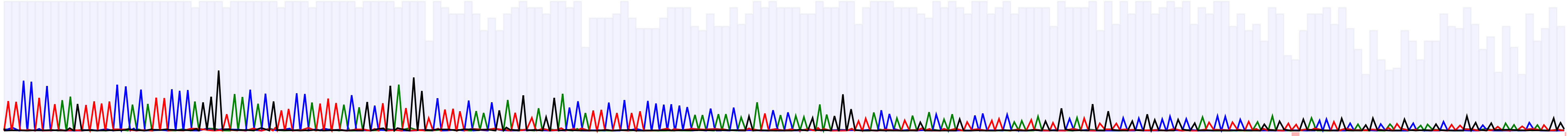

TTCCTCTAAGTTTTCCACATTCCCAGGGTAACACGTTCCATTTACAGCTGATGGTCTTTCAACGATGTAGGACCATTCTCTTCCCCCCAACAACAGATCACAAGAAGGGTTACCATAGACAAGTCCTTCAATAGTGCATGTGTCGCGGCCGCGATCCTCTAGAGTTGACCTGCAGGCATGCAAGCTTGGCGTAATCATGG

aligned sequence ANP799_PREMIX_ANP799_13 (ANP799_PREMIX_ANP799_13.ab1)

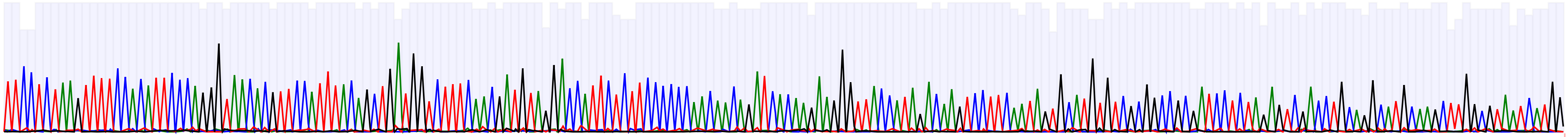

TTCCTCTAAGTTTTCCACATTCCCAGGGTAACACGTTCCATTTACAGCTGATGGTCTTTCAACGATGTAGGACCATTCTCTTCCCCCCAACAACAGATCACAAGAAGGGTTACCATAGACAAGTCCTTCAATAGTGCATGTGTCGCGGCCGCGATCCTCTAGAGTCGACCTGCAGGCATGCAAGCTTGGCGTAATCATGG

aligned sequence ANP811_PREMIX_ANP811_25 (ANP811_PREMIX_ANP811_25.ab1)

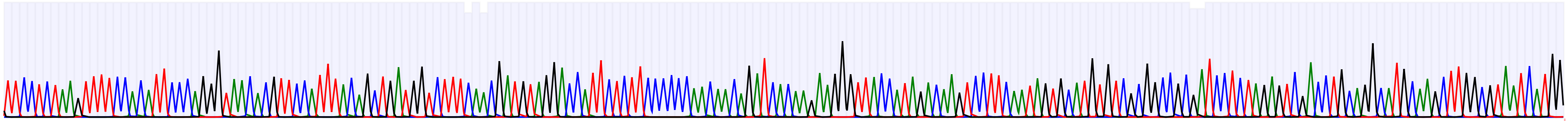

TTCCTCTAAGTTTTCCACATTCCCAGGGTAACACGTTCCATTTACAGCTGATGGTCTTTCAACGATGTAGGACCATTCTCTTCCCCCCAACAACAGATCACAAGAAGGGTTACCATAGACAAGTCCTTCAATAGTGCATGTGTCGCGGCCGCGATCCTCTAGAGTCGACCTGCAGGCATGCAAGCTTGGCGTAATCATGG

aligned sequence ANP814_PREMIX_ANP814_28 (ANP814_PREMIX_ANP814_28.ab1)

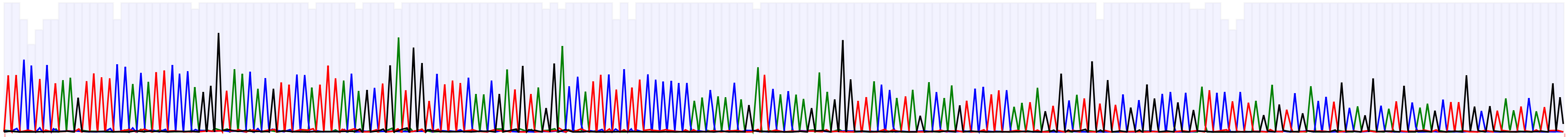

TTCCTCTAAGTTTTCCACATTCCCAGGGTAACACGTTCCATTTACAGCTGATGGTCTTTCAACGATGTAGGACCATTCTCTTCCCCCCAACAACAGATCACAAGAAGGGTTACCATAGACAAGTCCTTCAATAGTGCATGTGTCGCGGCCGCGATCCTCTAGAGTCGACCTGCAGGCATGCAAGCTTGGCGTAATCATGG

aligned sequence ANP804_PREMIX_ANP804_18 (ANP804_PREMIX_ANP804_18.ab1)

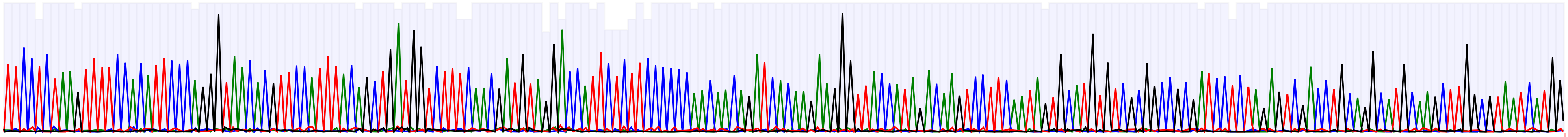

TTCCTCTAAGTTTTCCACATTCCCAGGGTAACACGTTCCATTTACAGCTGATGGTCTTTCAACGATGTAGGACCATTCTCTTCCCCCCAACAACAGATCACAAGAAGGGTTACCATAGACAAGTCCTTCAATAGTGCATGTGTCGCGGCCGCGATCCTCTAGAGTCGACCTGCAGGCATGCAAGCTTGGCGTAATCATGG

aligned sequence ANP812_PREMIX_ANP812_26 (ANP812_PREMIX_ANP812_26.ab1)

Template Alignment: Run 2 Assembly Only

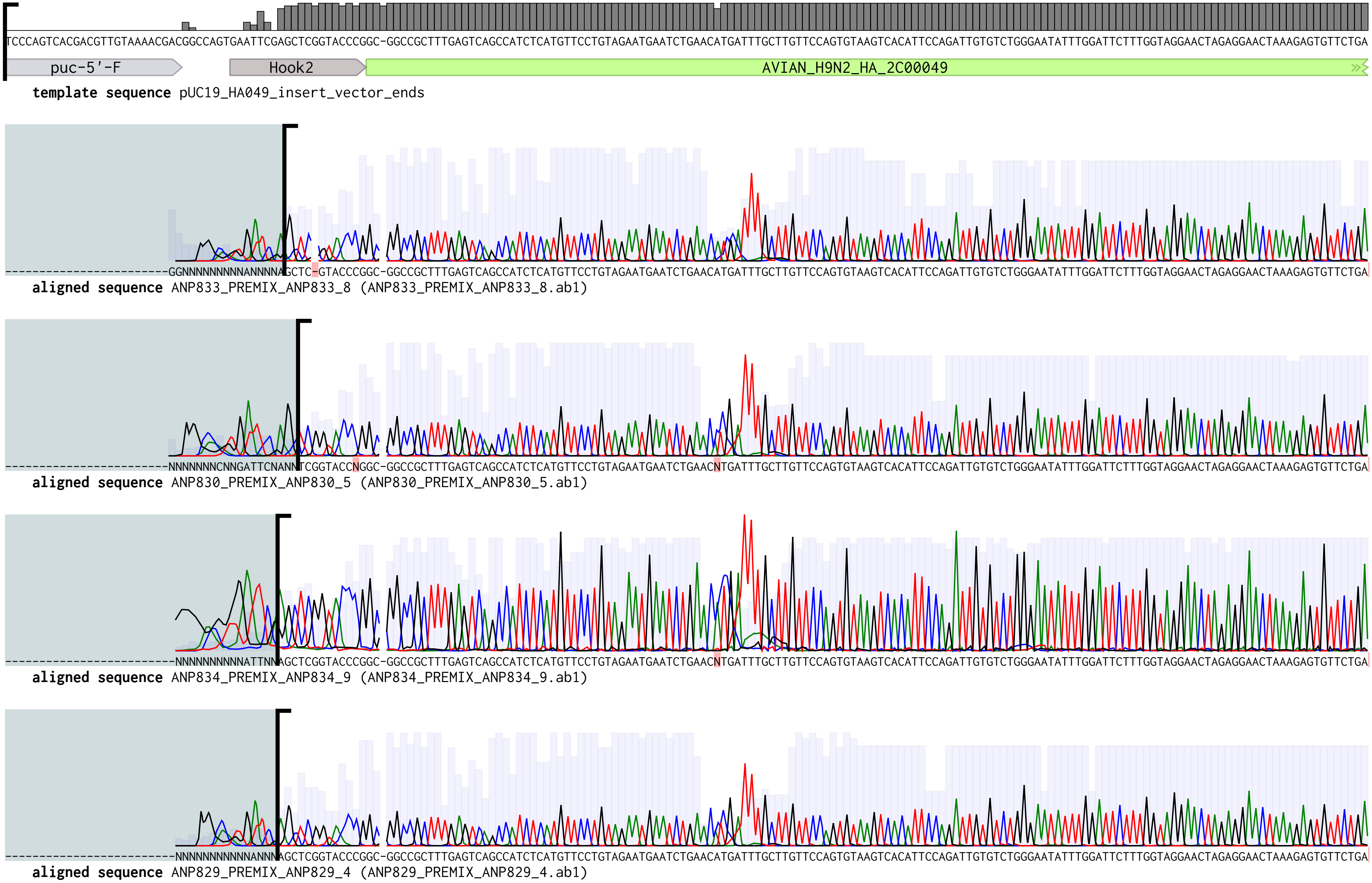

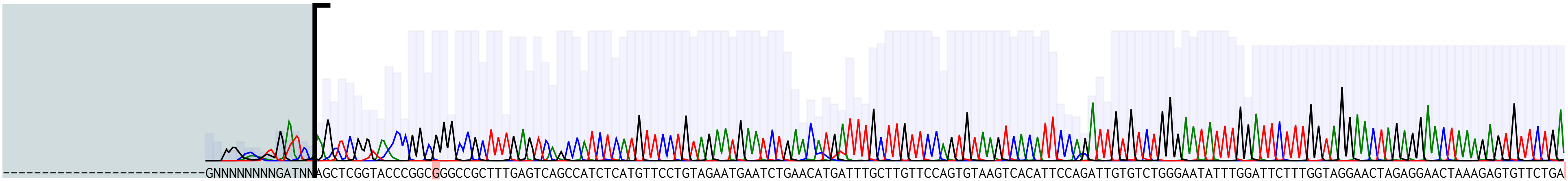

aligned sequence ANP827_PREMIX_ANP827_2 (ANP827_PREMIX_ANP827_2.ab1)

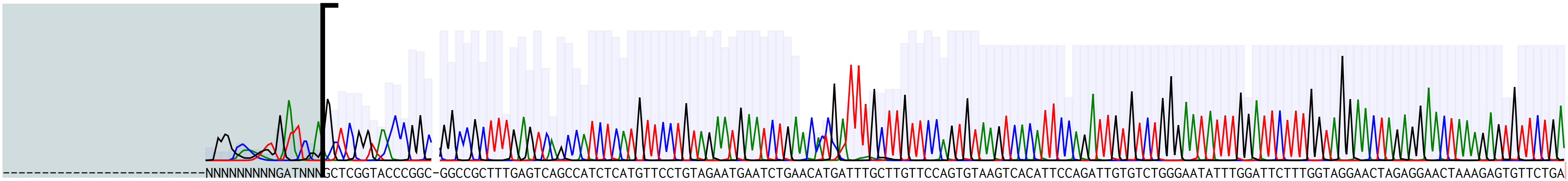

aligned sequence ANP831_PREMIX_ANP831_6 (ANP831_PREMIX_ANP831_6.ab1)

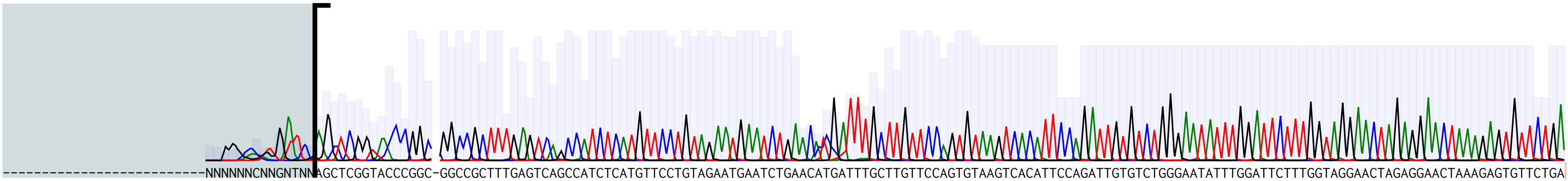

aligned sequence ANP835_PREMIX_ANP835_10 (ANP835_PREMIX_ANP835_10.ab1)

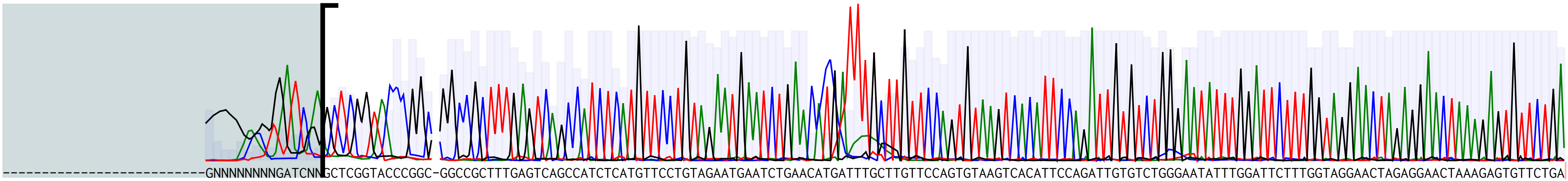

aligned sequence ANP832_PREMIX_ANP832_7 (ANP832_PREMIX_ANP832_7.ab1)

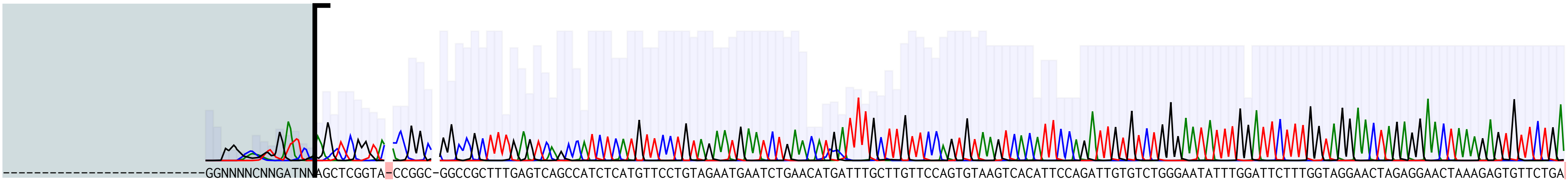

aligned sequence ANP828_PREMIX_ANP828_3 (ANP828_PREMIX_ANP828_3.ab1)

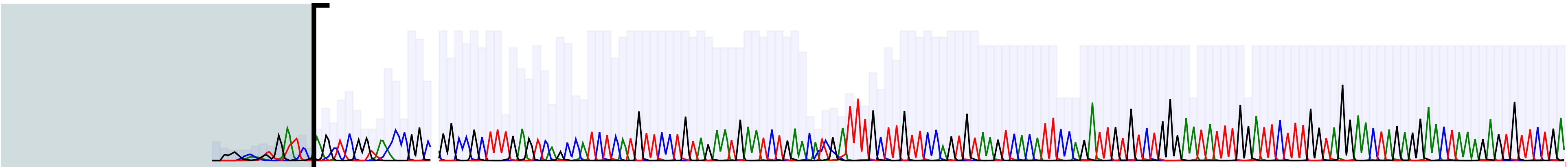

---------------------------NNNNNNNNNNNNCNAGCTCGGTACCCGGC-GGCCGCTTTGAGTCAGCCATCTCATGTTCCTGTAGAATGAATCTGAACATGATTTGCTTGTTCCAGTGTAAGTCACATTCCAGATTGTGTCTGGGAATATTTGGATTCTTTGGTAGGAACTAGAGGAACTAAAGAGTGTTCTGA

aligned sequence ANP826_PREMIX_ANP826_1 (ANP826_PREMIX_ANP826_1.ab1)

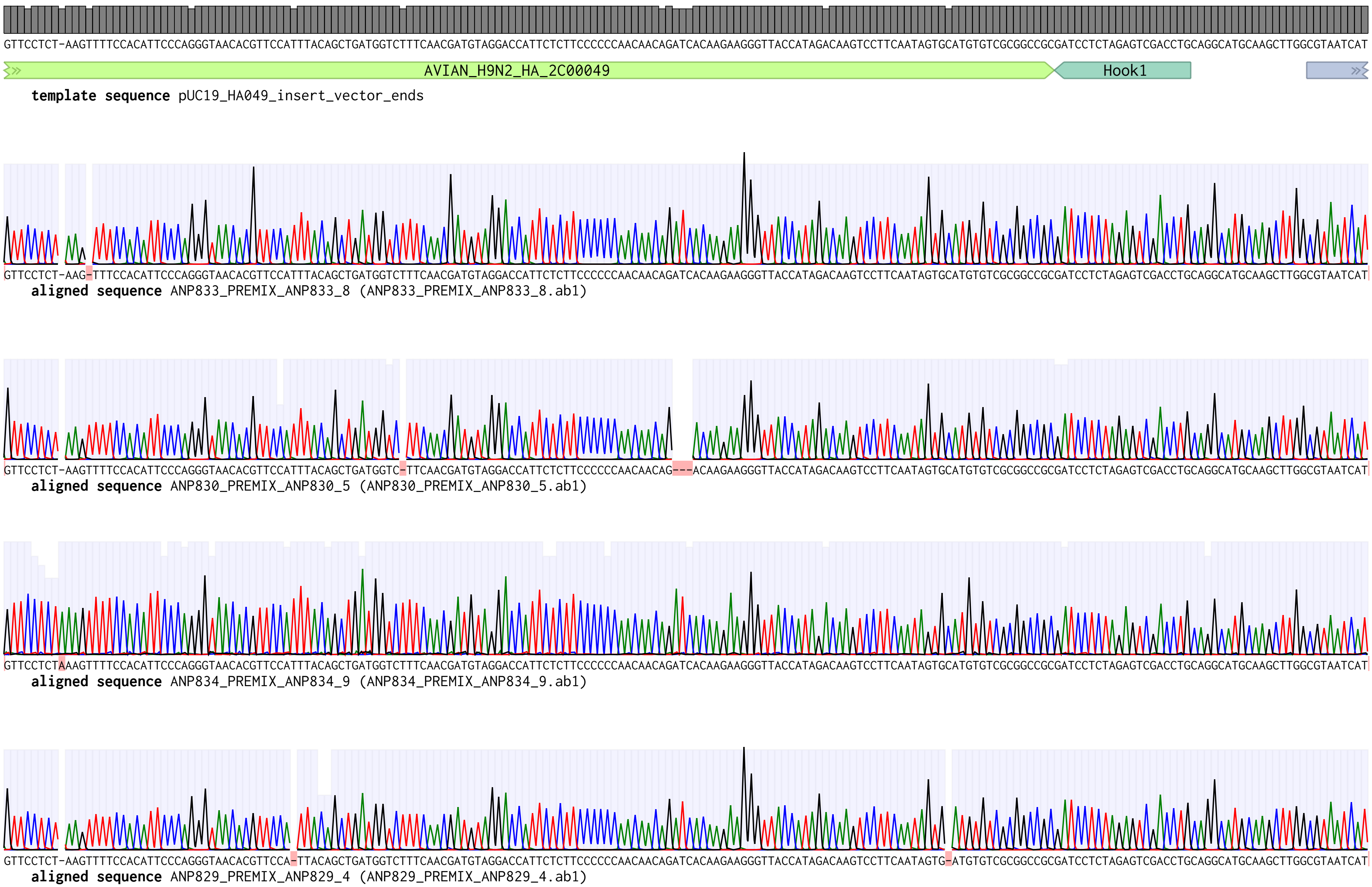
GTTCCTCT-AAGTTTTCCACATTCCCAGGGTAACACGTTCCATTTACAGCTGATGGTCTTTCAACGATGTAGGACCATTCTCTTCCCCCCAACAACAGATCACAAGAAGGGTTACCATAGACAAGTCCTTCAATAGTGCATGTGTCGCGGCCGCGATCCTCTAGAGTCGACCTGCAGGCATGCAAGCTTGGCGTAATCAT
AVIAN_H9N2_HA_2C00049
Hook1
template sequence pUC19_HA049_insert_vector_ends
GTTCCTCT-AAG-TTTCCACATTCCCAGGGTAACACGTTCCATTTACAGCTGATGGTCTTTCAACGATGTAGGACCATTCTCTTCCCCCCAACAACAGATCACAAGAAGGGTTACCATAGACAAGTCCTTCAATAGTGCATGTGTCGCGGCCGCGATCCTCTAGAGTCGACCTGCAGGCATGCAAGCTTGGCGTAATCAT
aligned sequence ANP833_PREMIX_ANP833_8 (ANP833_PREMIX_ANP833_8.ab1)
GTTCCTCT-AAGTTTTCCACATTCCCAGGGTAACACGTTCCATTTACAGCTGATGGTC-TTCAACGATGTAGGACCATTCTCTTCCCCCCAACAACAG---ACAAGAAGGGTTACCATAGACAAGTCCTTCAATAGTGCATGTGTCGCGGCCGCGATCCTCTAGAGTCGACCTGCAGGCATGCAAGCTTGGCGTAATCAT
aligned sequence ANP830_PREMIX_ANP830_5 (ANP830_PREMIX_ANP830_5.ab1)
GTTCCTCTAAAGTTTTCCACATTCCCAGGGTAACACGTTCCATTTACAGCTGATGGTCTTTCAACGATGTAGGACCATTCTCTTCCCCCCAACAACAGATCACAAGAAGGGTTACCATAGACAAGTCCTTCAATAGTGCATGTGTCGCGGCCGCGATCCTCTAGAGTCGACCTGCAGGCATGCAAGCTTGGCGTAATCAT
aligned sequence ANP834_PREMIX_ANP834_9 (ANP834_PREMIX_ANP834_9.ab1)
GTTCCTCT-AAGTTTTCCACATTCCCAGGGTAACACGTTCCA-TTACAGCTGATGGTCTTTCAACGATGTAGGACCATTCTCTTCCCCCCAACAACAGATCACAAGAAGGGTTACCATAGACAAGTCCTTCAATAGTG-ATGTGTCGCGGCCGCGATCCTCTAGAGTCGACCTGCAGGCATGCAAGCTTGGCGTAATCAT
aligned sequence ANP829_PREMIX_ANP829_4 (ANP829_PREMIX_ANP829_4.ab1)

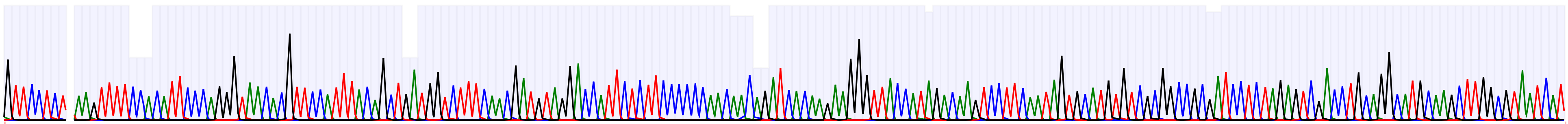

GTTCCTCT-AAGTTTTCCACATTCCCAGGGTAACACGTTCCATTTACAGCTGATGGTCTTTCAACGATGTAGGACCATTCTCTTCCCCCCAACAACAGATCACAAGAAGGGTTACCATAGACAAGTCCTTCAATAGTGCATGTGTCGCGGCCGCGATCCTCTAGAGTCGACCTGCAGGCATGCAAGCTTGGCGTAATCAT

aligned sequence ANP827_PREMIX_ANP827_2 (ANP827_PREMIX_ANP827_2.ab1)

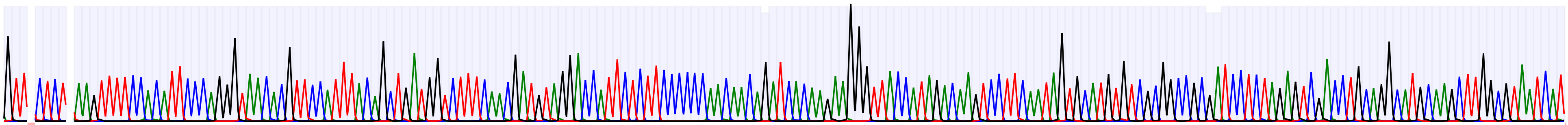

GTT-CTCT-AAGTTTTCCACATTCCCAGGGTAACACGTTCCATTTACAGCTGATGGTCTTTCAACGATGTAGGACCATTCTCTTCCCCCCAACAACAGATCACAAGAAGGGTTACCATAGACAAGTCCTTCAATAGTGCATGTGTCGCGGCCGCGATCCTCTAGAGTCGACCTGCAGGCATGCAAGCTTGGCGTAATCAT

aligned sequence ANP831_PREMIX_ANP831_6 (ANP831_PREMIX_ANP831_6.ab1)

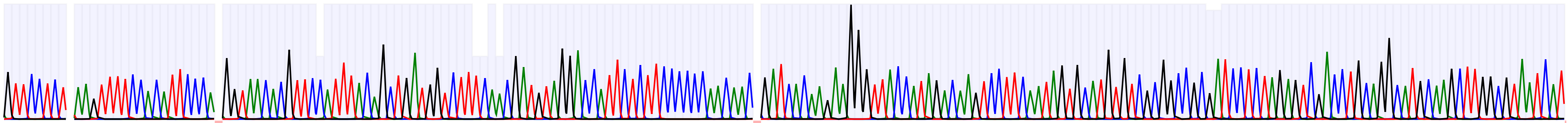

GTTCCTCT-AAGTTTTCCACATTCCCA-GGTAACACGTTCCATTTACAGCTGATGGTCTTTCAACGATGTAGGACCATTCTCTTCCCCCCAACAAC-GATCACAAGAAGGGTTACCATAGACAAGTCCTTCAATAGTGCATGTGTCGCGGCCGCGATCCTCTAGAGTCGACCTGCAGGCATGCAAGCTTGGCGTAATCAT

aligned sequence ANP835_PREMIX_ANP835_10 (ANP835_PREMIX_ANP835_10.ab1)

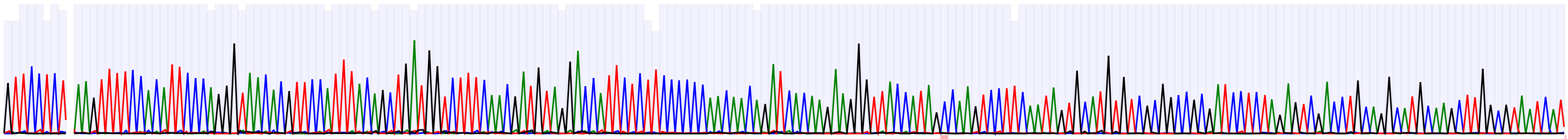

GTTCCTCT-AAGTTTTCCACATTCCCAGGGTAACACGTTCCATTTACAGCTGATGGTCTTTCAACGATGTAGGACCATTCTCTTCCCCCCAACAACAGATCACAAGAAGGGTTACCATAGCCAAGTCCTTCAATAGTGCATGTGTCGCGGCCGCGATCCTCTAGAGTCGACCTGCAGGCATGCAAGCTTGGCGTAATCAT

aligned sequence ANP832_PREMIX_ANP832_7 (ANP832_PREMIX_ANP832_7.ab1)

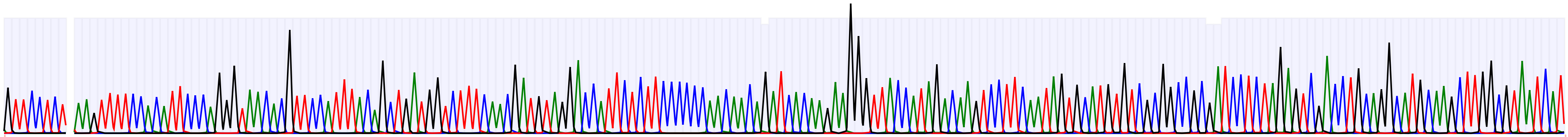

GTTCCTCT-AAGTTTTCCACATTCCCAGGGTAACACGTTCCATTTACAGCTGATGGTCTTTCAACGATGTAGGACCATTCTCTTCCCCCCAACAACAGATCACAAGAAGGGTTACCATAGACAAGTCCTTCAATAGTGCATGTGTCGCGGCCGCGATCCTCTAGAGTCGACCTGCAGGCATGCAAGCTTGGCGTAATCAT

aligned sequence ANP828_PREMIX_ANP828_3 (ANP828_PREMIX_ANP828_3.ab1)

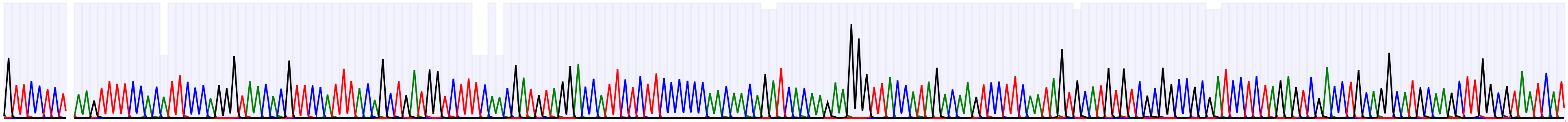

GTTCCTCT-AAGTTTTCCACATTCCCAGGGTAACACGTTCCATTTACAGCTGATGGTCTTTCAACGATGTAGGACCATTCTCTTCCCCCCAACAACAGATCACAAGAAGGGTTACCATAGACAAGTCCTTCAATAGTGCATGTGTCGCGGCCGCGATCCTCTAGAGTCGACCTGCAGGCATGCAAGCTTGGCGTAATCAT

aligned sequence ANP826_PREMIX_ANP826_1 (ANP826_PREMIX_ANP826_1.ab1)

Template Alignment: Run 2 error correction

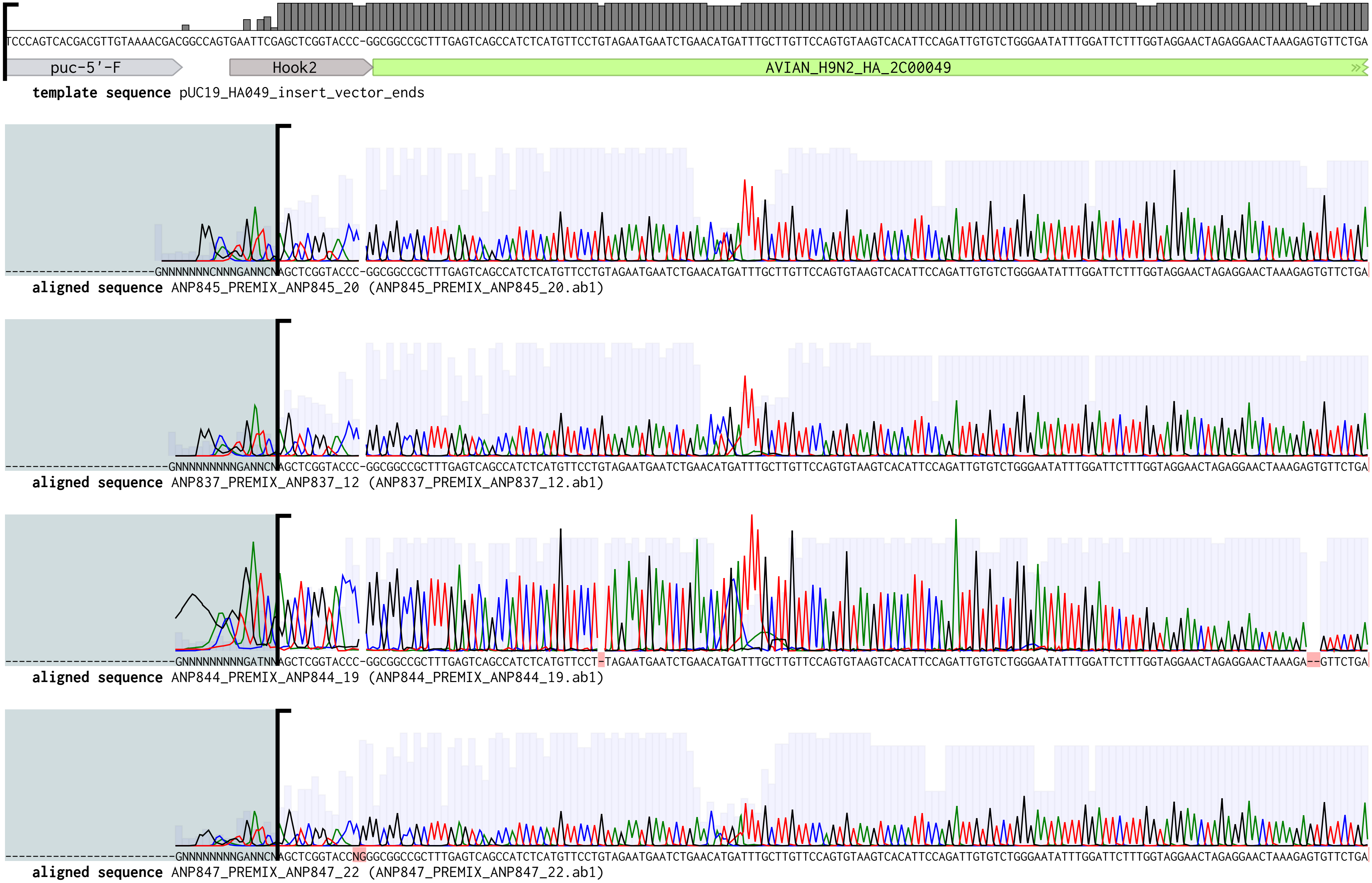

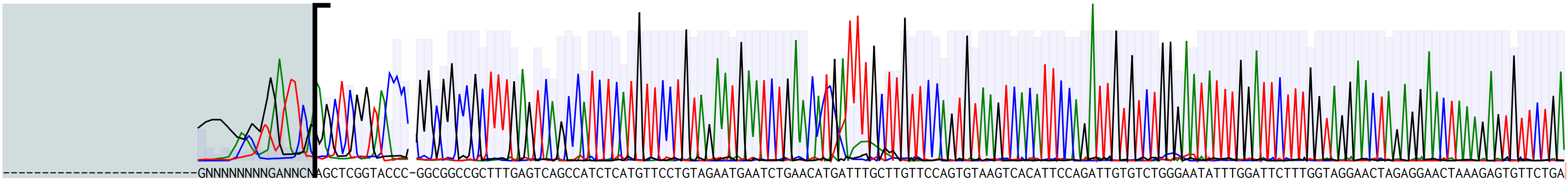

aligned sequence ANP843_PREMIX_ANP843_18 (ANP843_PREMIX_ANP843_18.ab1)

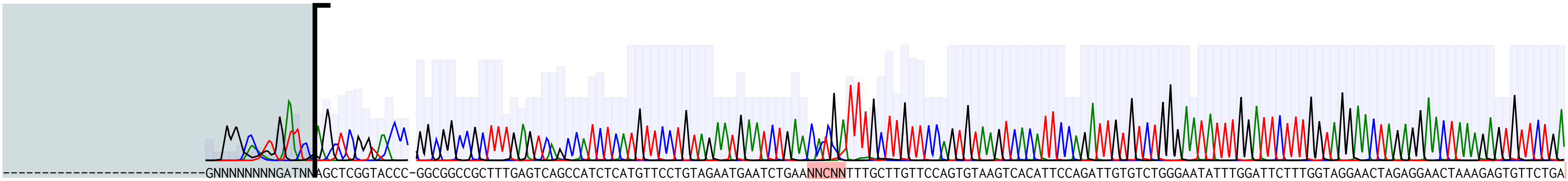

aligned sequence ANP838_PREMIX_ANP838_13 (ANP838_PREMIX_ANP838_13.ab1)

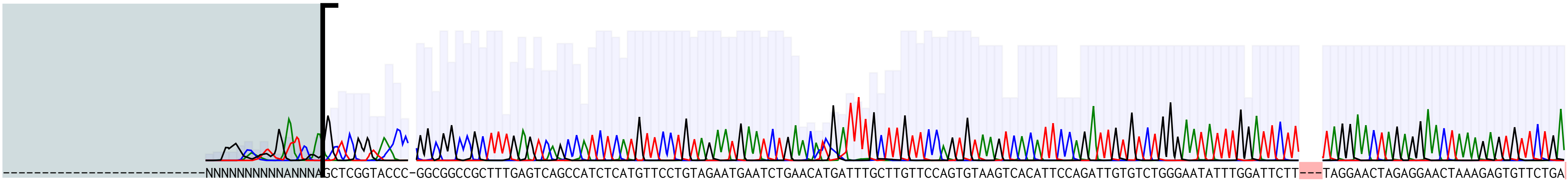

aligned sequence ANP848_PREMIX_ANP848_23 (ANP848_PREMIX_ANP848_23.ab1)

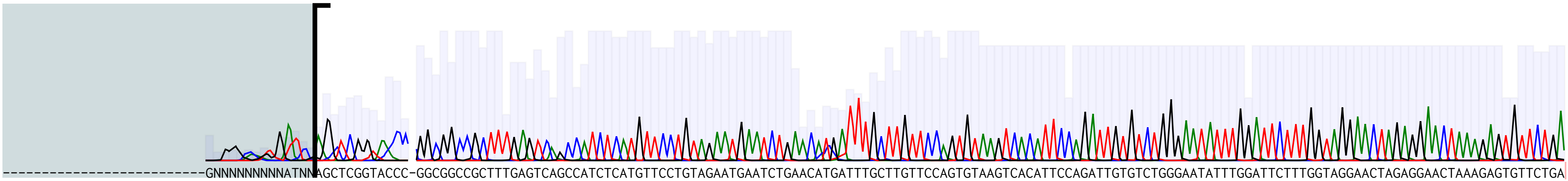

aligned sequence ANP850_PREMIX_ANP850_25 (ANP850_PREMIX_ANP850_25.ab1)

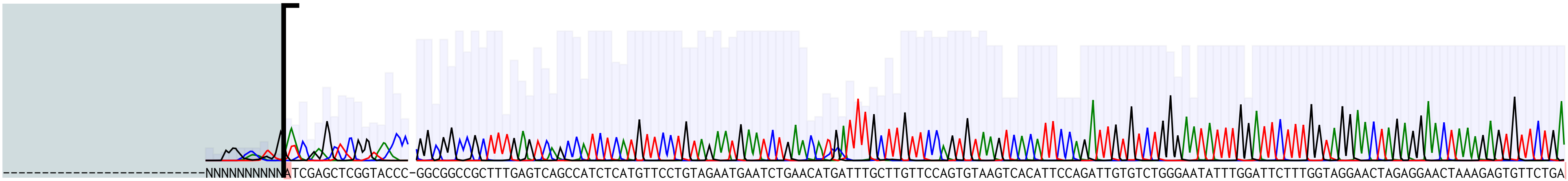

aligned sequence ANP842_PREMIX_ANP842_17 (ANP842_PREMIX_ANP842_17.ab1)

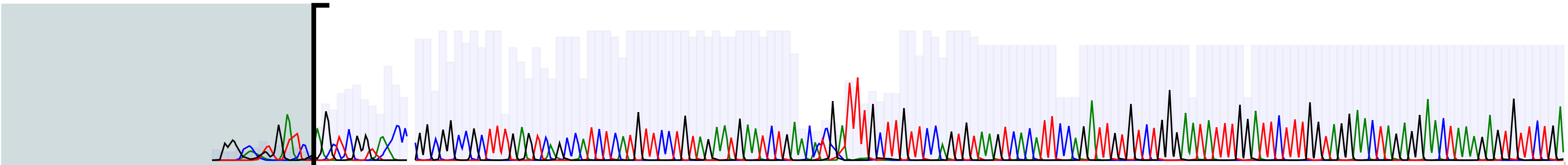

----------------------------NNNNNNNNNNNNNNAGCTCGGTACCC-GGCGGCCGCTTTGAGTCAGCCATCTCATGTTCCTGTAGAATGAATCTGAACATGATTTGCTTGTTCCAGTGTAAGTCACATTCCAGATTGTGTCTGGGAATATTTGGATTCTTTGGTAGGAACTAGAGGAACTAAAGAGTGTTCTGA

aligned sequence ANP839_PREMIX_ANP839_14 (ANP839_PREMIX_ANP839_14.ab1)

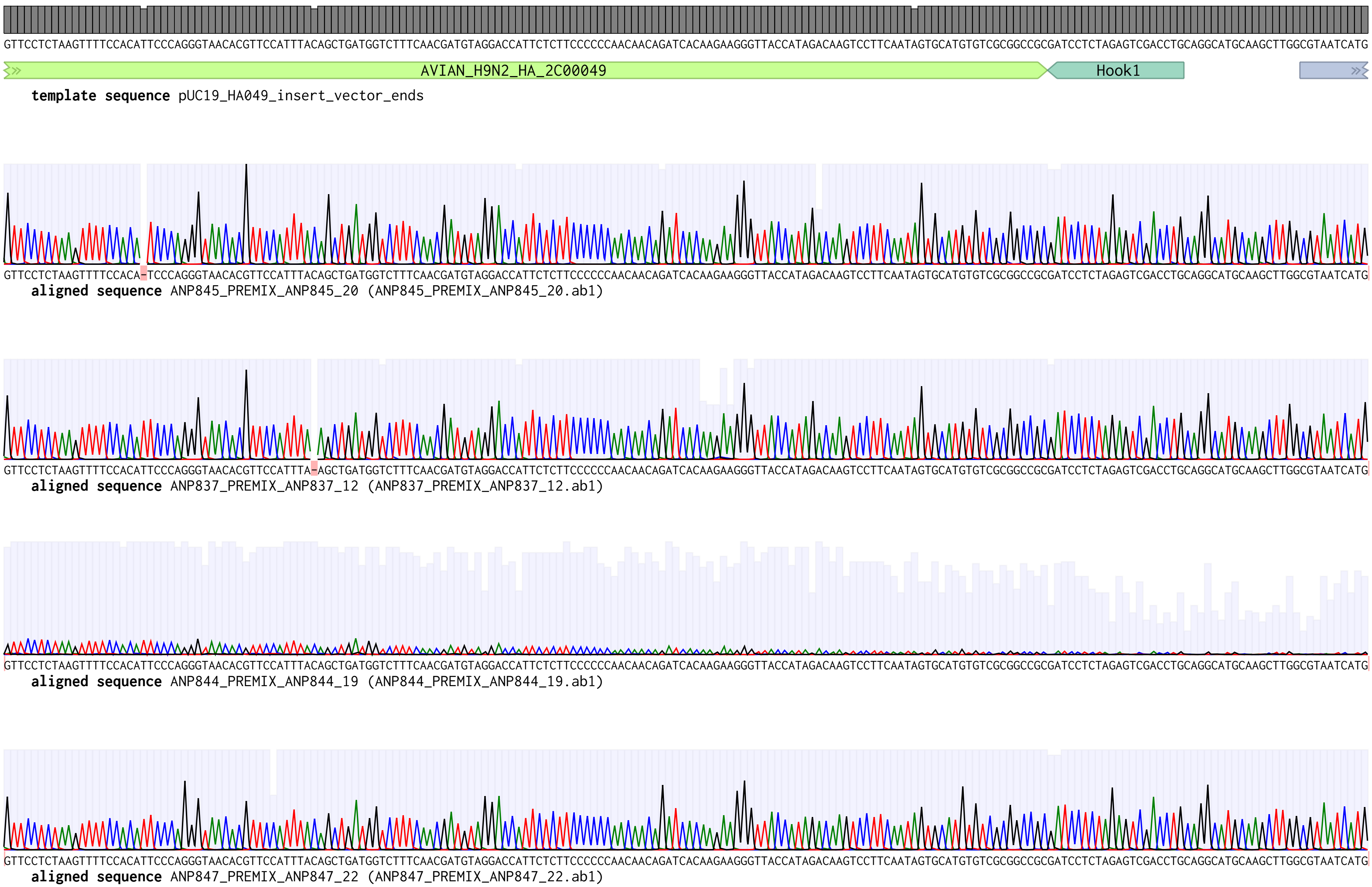
AVIAN_H9N2_HA_2C00049
Hook1
template sequence pUC19_HA049_insert_vector_ends
aligned sequence ANP845_PREMIX_ANP845_20 (ANP845_PREMIX_ANP845_20.ab1)
aligned sequence ANP837_PREMIX_ANP837_12 (ANP837_PREMIX_ANP837_12.ab1)
aligned sequence ANP844_PREMIX_ANP844_19 (ANP844_PREMIX_ANP844_19.ab1)
aligned sequence ANP847_PREMIX_ANP847_22 (ANP847_PREMIX_ANP847_22.ab1)

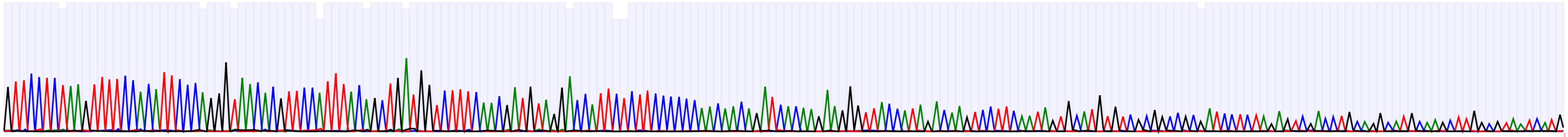

GTTCCTCTAAGTTTTCCACATTCCCAGGGTAACACGTTCCATTTACAGCTGATGGTCTTTCAACGATGTAGGACCATTCTCTTCCCCCCAACAACAGATCACAAGAAGGGTTACCATAGACAAGTCCTTCAATAGTGCATGTGTCGCGGCCGCGATCCTCTAGAGTCGACCTGCAGGCATGCAAGCTTGGCGTAATCATG

aligned sequence ANP843_PREMIX_ANP843_18 (ANP843_PREMIX_ANP843_18.ab1)

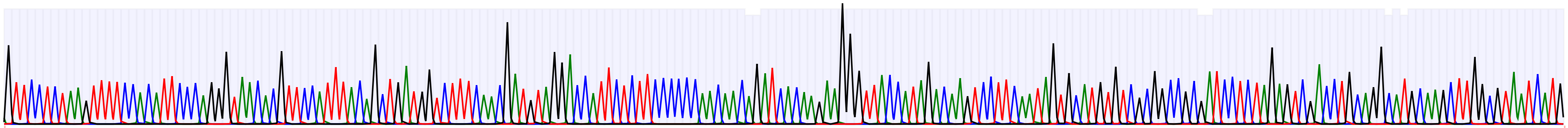

GTTCCTCTAAGTTTTCCACATTCCCAGGGTAACACGTTCCATTTACAGCTGATGGTCTTTCAACGATGTAGGACCATTCTCTTCCCCCCAACAACAGATCACAAGAAGGGTTACCATAGACAAGTCCTTCAATAGTGCATGTGTCGCGGCCGCGATCCTCTAGAGTCGACCTGCAGGCATGCAAGCTTGGCGTAATCATG

aligned sequence ANP838_PREMIX_ANP838_13 (ANP838_PREMIX_ANP838_13.ab1)

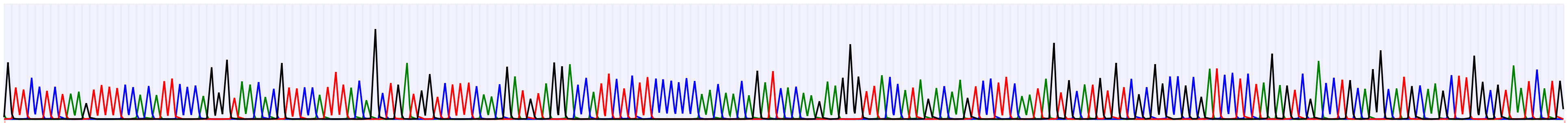

GTTCCTCTAAGTTTTCCACATTCCCAGGGTAACACGTTCCATTTACAGCTGATGGTCTTTCAACGATGTAGGACCATTCTCTTCCCCCCAACAACAGATCACAAGAAGGGTTACCATAGACAAGTCCTTCAATAGTGCATGTGTCGCGGCCGCGATCCTCTAGAGTCGACCTGCAGGCATGCAAGCTTGGCGTAATCATG

aligned sequence ANP848_PREMIX_ANP848_23 (ANP848_PREMIX_ANP848_23.ab1)

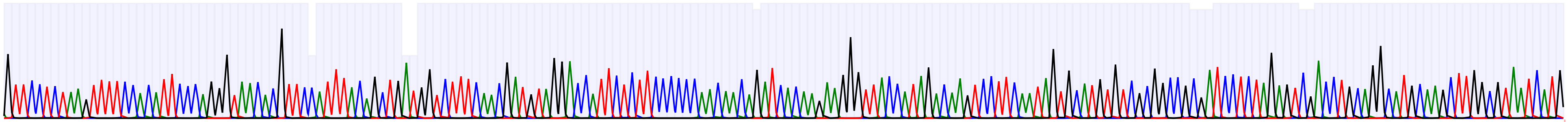

GTTCCTCTAAGTTTTCCACATTCCCAGGGTAACACGTTCCATTTACAGCTGATGGTCTTTCAACGATGTAGGACCATTCTCTTCCCCCCAACAACAGATCACAAGAAGGGTTACCATAGACAAGTCCTTCAATAGTGCATGTGTCGCGGCCGCGATCCTCTAGAGTCGACCTGCAGGCATGCAAGCTTGGCGTAATCATG

aligned sequence ANP850_PREMIX_ANP850_25 (ANP850_PREMIX_ANP850_25.ab1)

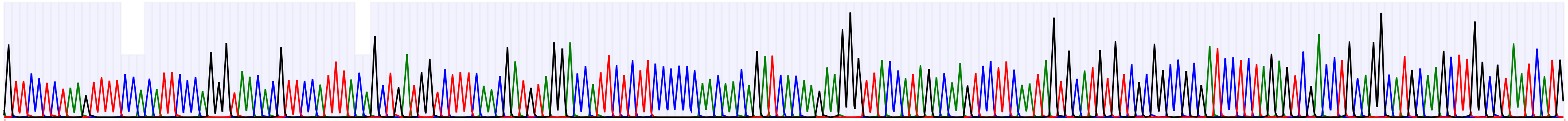

GTTCCTCTAAGTTTTCCACATTCCCAGGGTAACACGTTCCATTTACAGCTGATGGTCTTTCAACGATGTAGGACCATTCTCTTCCCCCCAACAACAGATCACAAGAAGGGTTACCATAGACAAGTCCTTCAATAGTGCATGTGTCGCGGCCGCGATCCTCTAGAGTCGACCTGCAGGCATGCAAGCTTGGCGTAATCATG

aligned sequence ANP842_PREMIX_ANP842_17 (ANP842_PREMIX_ANP842_17.ab1)

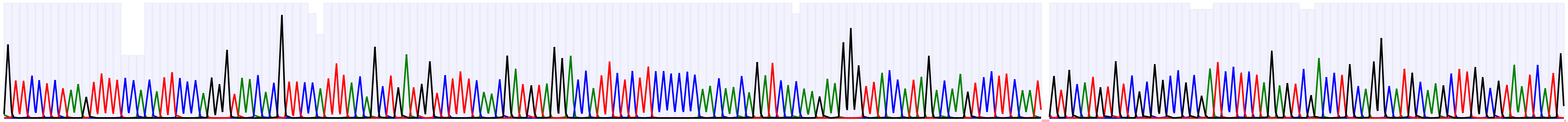

GTTCCTCTAAGTTTTCCACATTCCCAGGGTAACACGTTCCATTTACAGCTGATGGTCTTTCAACGATGTAGGACCATTCTCTTCCCCCCAACAACAGATCACAAGAAGGGTTACCATAGACAAGTCCTTCAAT-GTGCATGTGTCGCGGCCGCGATCCTCTAGAGTCGACCTGCAGGCATGCAAGCTTGGCGTAATCATG

aligned sequence ANP839_PREMIX_ANP839_14 (ANP839_PREMIX_ANP839_14.ab1)

Template Alignment: Run 3 Assembly Only

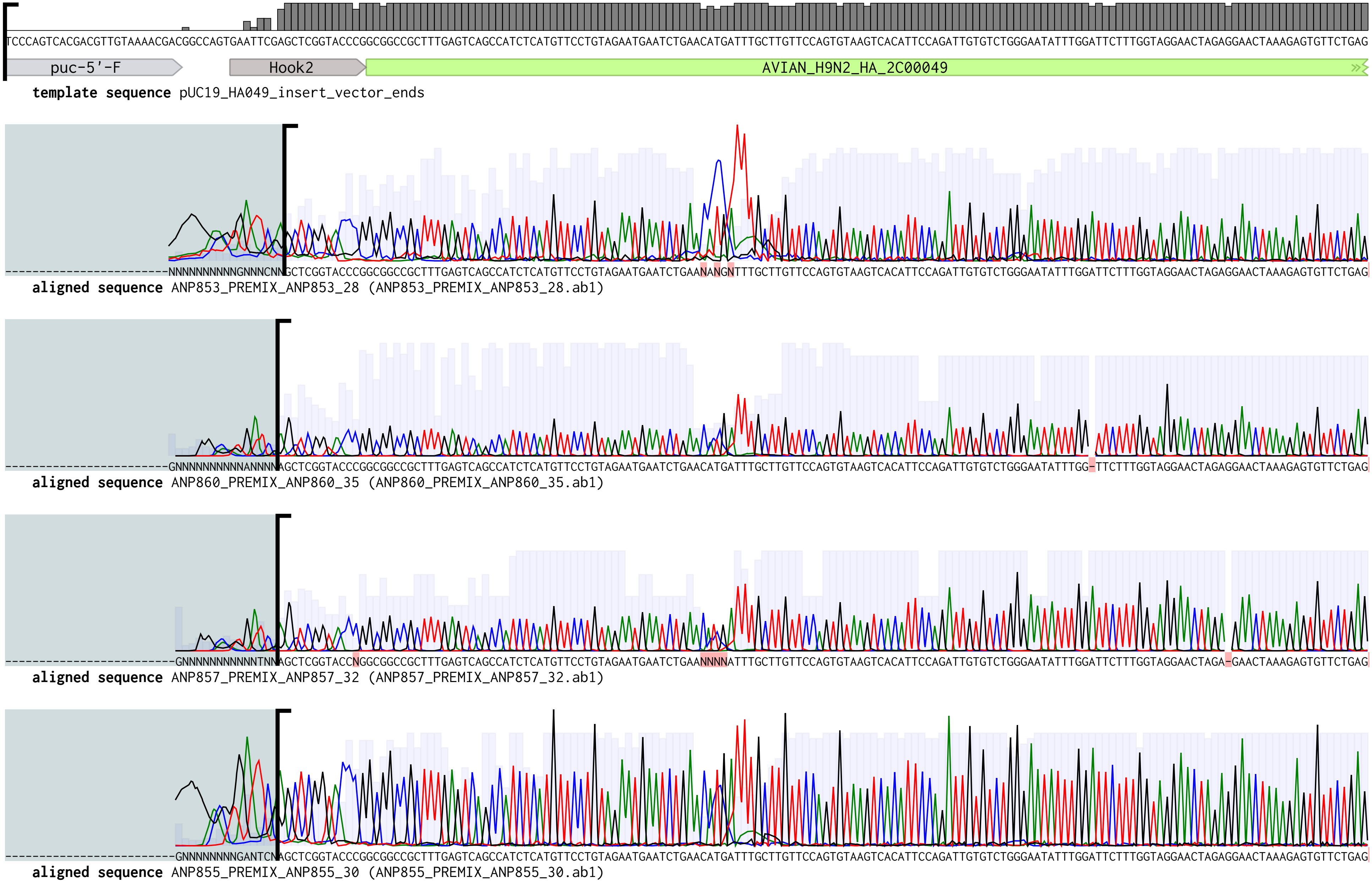

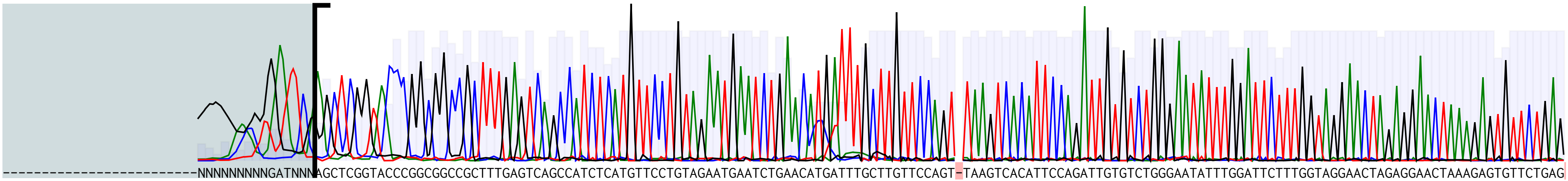

aligned sequence ANP858_PREMIX_ANP858_33 (ANP858_PREMIX_ANP858_33.ab1)

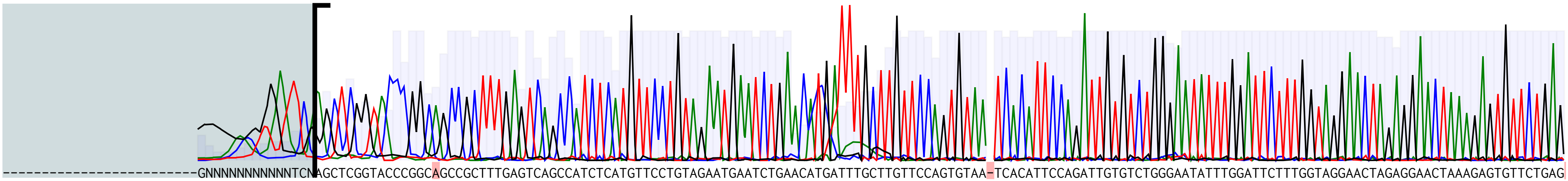

aligned sequence ANP859_PREMIX_ANP859_34 (ANP859_PREMIX_ANP859_34.ab1)

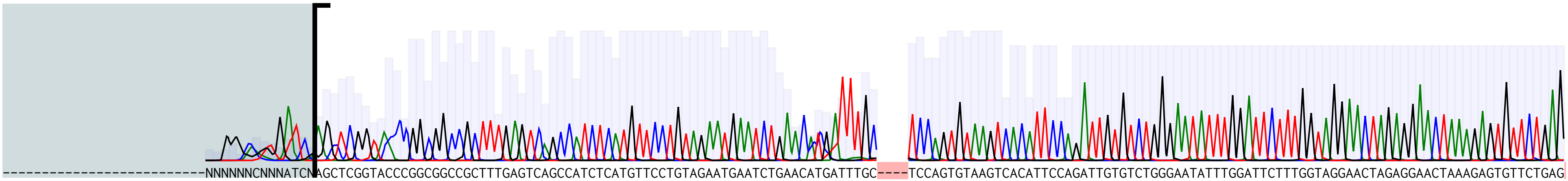

aligned sequence ANP856_PREMIX_ANP856_31 (ANP856_PREMIX_ANP856_31.ab1)

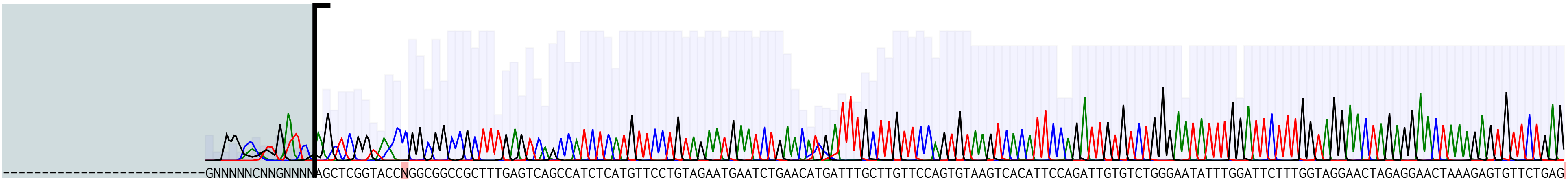

aligned sequence ANP852_PREMIX_ANP852_27 (ANP852_PREMIX_ANP852_27.ab1)

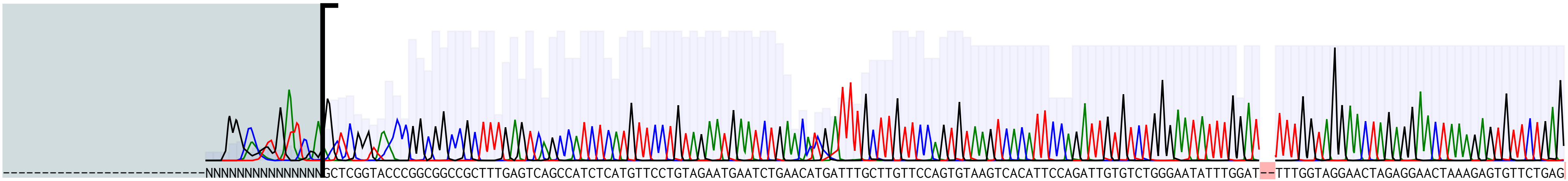

aligned sequence ANP854_PREMIX_ANP854_29 (ANP854_PREMIX_ANP854_29.ab1)

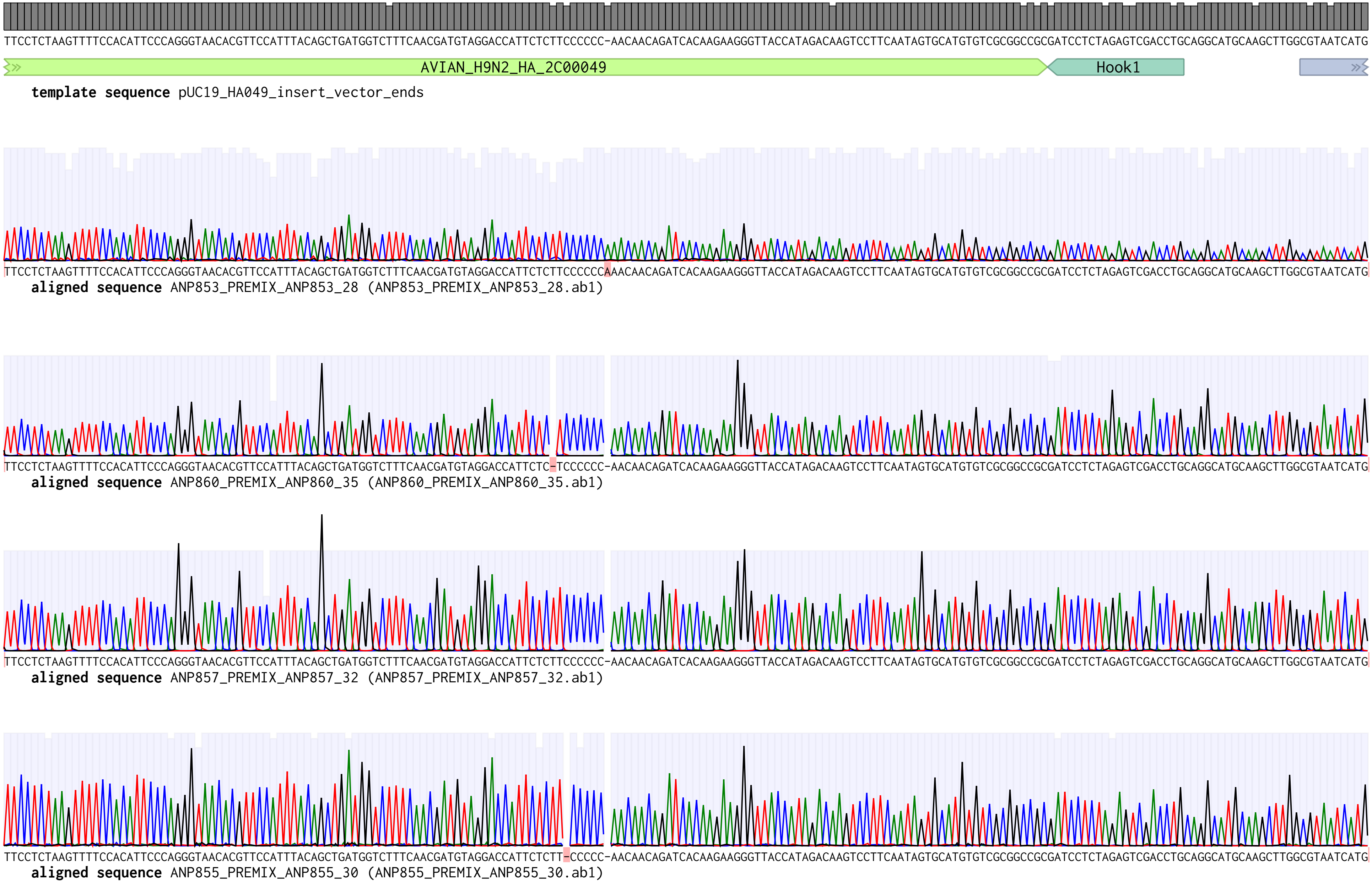
TTCCTCTAAGTTTTCCACATTCCCAGGGTAACACGTTCCATTTACAGCTGATGGTCTTTCAACGATGTAGGACCATTCTCTTCCCCCC-AACAACAGATCACAAGAAGGGTTACCATAGACAAGTCCTTCAATAGTGCATGTGTCGCGGCCGCGATCCTCTAGAGTCGACCTGCAGGCATGCAAGCTTGGCGTAATCATG
AVIAN_H9N2_HA_2C00049
Hook1
template sequence pUC19_HA049_insert_vector_ends
TTCCTCTAAGTTTTCCACATTCCCAGGGTAACACGTTCCATTTACAGCTGATGGTCTTTCAACGATGTAGGACCATTCTCTTCCCCCCAAACAACAGATCACAAGAAGGGTTACCATAGACAAGTCCTTCAATAGTGCATGTGTCGCGGCCGCGATCCTCTAGAGTCGACCTGCAGGCATGCAAGCTTGGCGTAATCATG
aligned sequence ANP853_PREMIX_ANP853_28 (ANP853_PREMIX_ANP853_28.ab1)
TTCCTCTAAGTTTTCCACATTCCCAGGGTAACACGTTCCATTTACAGCTGATGGTCTTTCAACGATGTAGGACCATTCTC-TCCCCCC-AACAACAGATCACAAGAAGGGTTACCATAGACAAGTCCTTCAATAGTGCATGTGTCGCGGCCGCGATCCTCTAGAGTCGACCTGCAGGCATGCAAGCTTGGCGTAATCATG
aligned sequence ANP860_PREMIX_ANP860_35 (ANP860_PREMIX_ANP860_35.ab1)
TTCCTCTAAGTTTTCCACATTCCCAGGGTAACACGTTCCATTTACAGCTGATGGTCTTTCAACGATGTAGGACCATTCTCTTCCCCCC-AACAACAGATCACAAGAAGGGTTACCATAGACAAGTCCTTCAATAGTGCATGTGTCGCGGCCGCGATCCTCTAGAGTCGACCTGCAGGCATGCAAGCTTGGCGTAATCATG
aligned sequence ANP857_PREMIX_ANP857_32 (ANP857_PREMIX_ANP857_32.ab1)
TTCCTCTAAGTTTTCCACATTCCCAGGGTAACACGTTCCATTTACAGCTGATGGTCTTTCAACGATGTAGGACCATTCTCTT-CCCCC-AACAACAGATCACAAGAAGGGTTACCATAGACAAGTCCTTCAATAGTGCATGTGTCGCGGCCGCGATCCTCTAGAGTCGACCTGCAGGCATGCAAGCTTGGCGTAATCATG
aligned sequence ANP855_PREMIX_ANP855_30 (ANP855_PREMIX_ANP855_30.ab1)

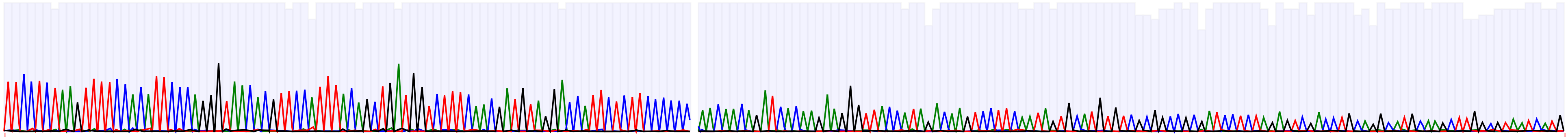

TTCCTCTAAGTTTTCCACATTCCCAGGGTAACACGTTCCATTTACAGCTGATGGTCTTTCAACGATGTAGGACCATTCTCTTCCCCCC-AACAACAGATCACAAGAAGGGTTACCATAGACAAGTCCTTCAATAGTGCATGTGTCGCGGCCGCGATCCTCTAGAGTCGACCTGCAGGCATGCAAGCTTGGCGTAATCATG

aligned sequence ANP858_PREMIX_ANP858_33 (ANP858_PREMIX_ANP858_33.ab1)

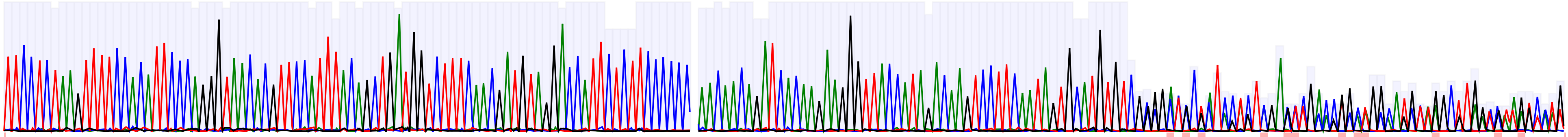

TTCCTCTAAGTTTTCCACATTCCCAGGGTAACACGTTCCATTTACAGCTGATGGTCTTTCAACGATGTAGGACCATTCTCTTCCCCCC-AACAACAGATCACAAGAAGGGTTACCATAGACAAGTCCTTCAATAGTGCATGTGTCGCGGACCCNATCCTCTCGACCCGACCGGNNGGCATGCAGGCTTGGCCTAGTCATG

aligned sequence ANP859_PREMIX_ANP859_34 (ANP859_PREMIX_ANP859_34.ab1)

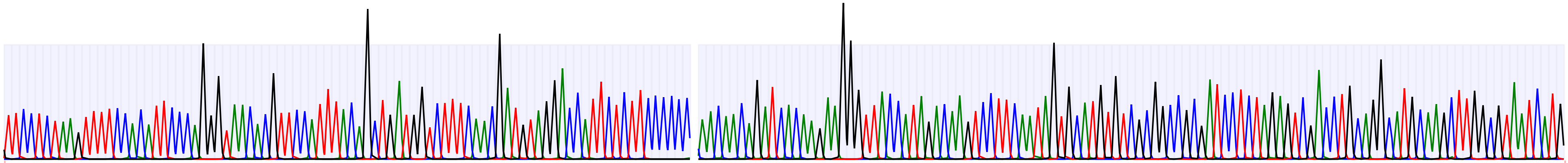

TTCCTCTAAGTTTTCCACATTCCCAGGGTAACACGTTCCATTTACAGCTGATGGTCTTTCAACGATGTAGGACCATTCTCTTCCCCCC-AACAACAGATCACAAGAAGGGTTACCATAGACAAGTCCTTCAATAGTGCATGTGTCGCGGCCGCGATCCTCTAGAGTCGACCTGCAGGCATGCAAGCTTGGCGTAATCATG

aligned sequence ANP856_PREMIX_ANP856_31 (ANP856_PREMIX_ANP856_31.ab1)

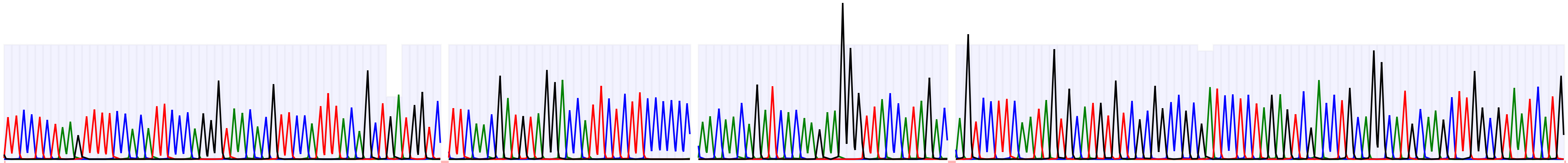

TTCCTCTAAGTTTTCCACATTCCCAGGGTAACACGTTCCATTTACAGCTGATGGTC-TTCAACGATGTAGGACCATTCTCTTCCCCCC-AACAACAGATCACAAGAAGGGTTACCATAGAC-AGTCCTTCAATAGTGCATGTGTCGCGGCCGCGATCCTCTAGAGTCGACCTGCAGGCATGCAAGCTTGGCGTAATCATG

aligned sequence ANP852_PREMIX_ANP852_27 (ANP852_PREMIX_ANP852_27.ab1)

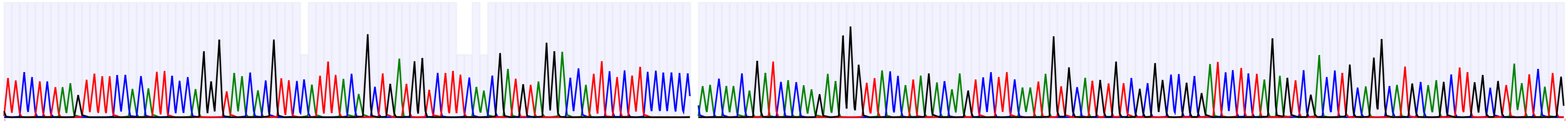

TTCCTCTAAGTTTTCCACATTCCCAGGGTAACACGTTCCATTTACAGCTGATGGTCTTTCAACGATGTAGGACCATTCTCTTCCCCCC-AACAACAGATCACAAGAAGGGTTACCATAGACAAGTCCTTCAATAGTGCATGTGTCGCGGCCGCGATCCTCTAGAGTCGACCTGCAGGCATGCAAGCTTGGCGTAATCATG

aligned sequence ANP854_PREMIX_ANP854_29 (ANP854_PREMIX_ANP854_29.ab1)

Template Alignment: Run 3 Error Correction

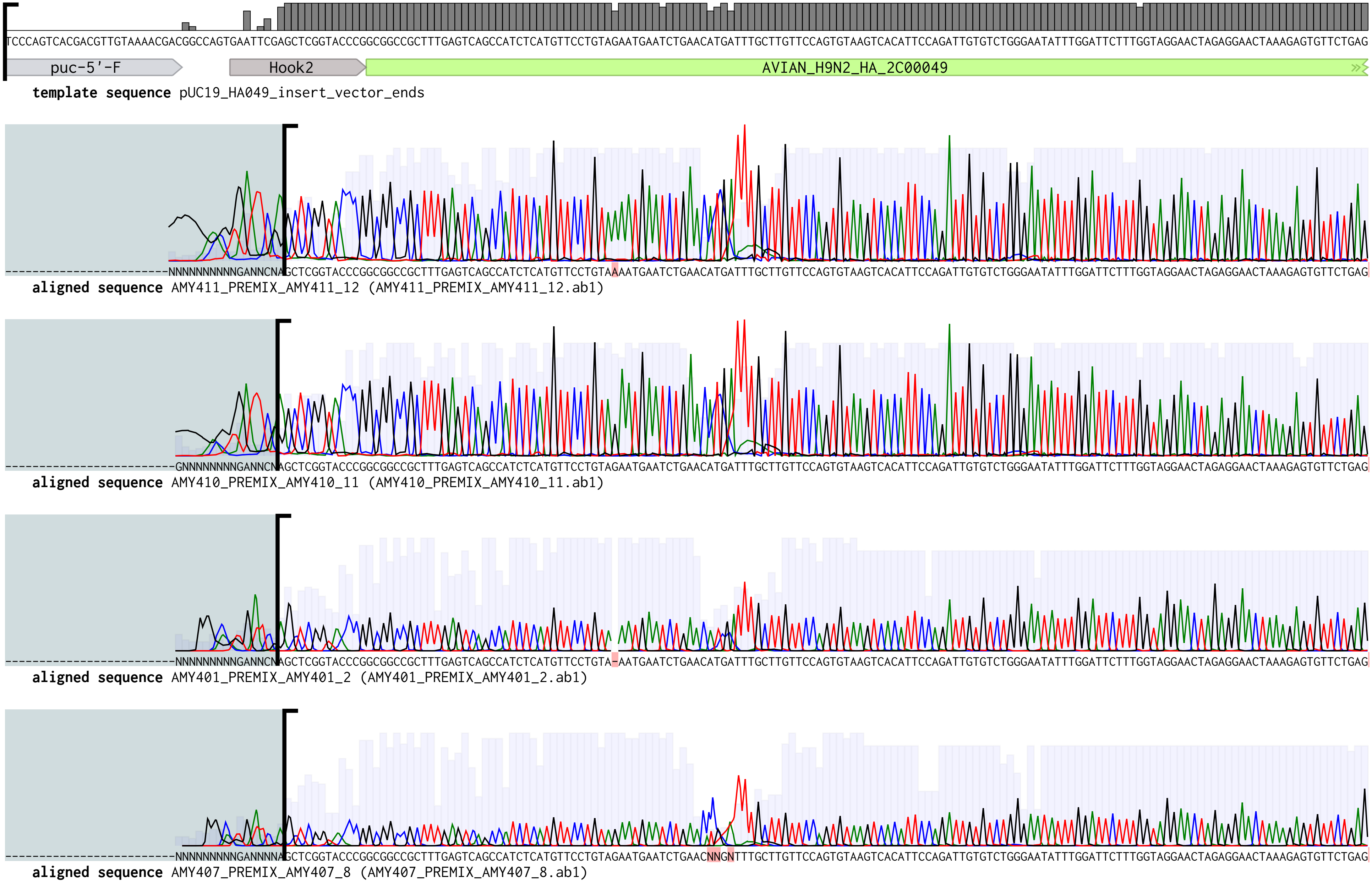

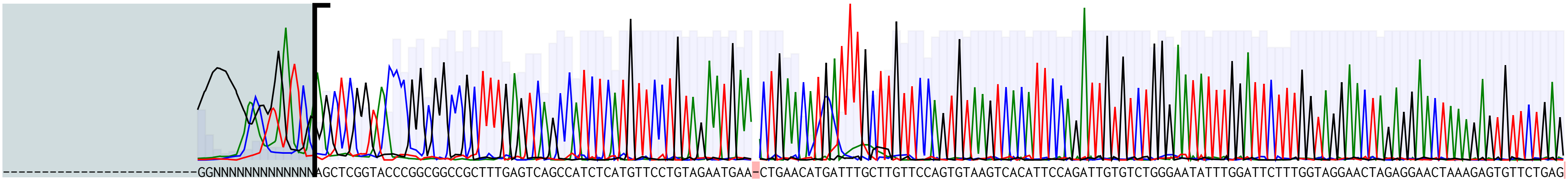

aligned sequence ANP862_PREMIX_ANP862_37 (ANP862_PREMIX_ANP862_37.ab1)

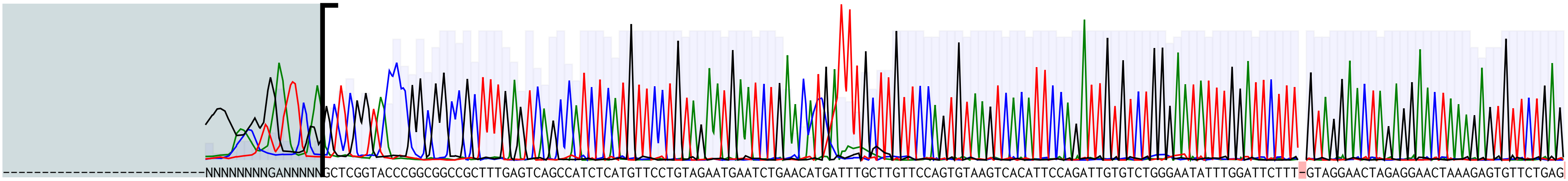

aligned sequence AMY403_PREMIX_AMY403_4 (AMY403_PREMIX_AMY403_4.ab1)

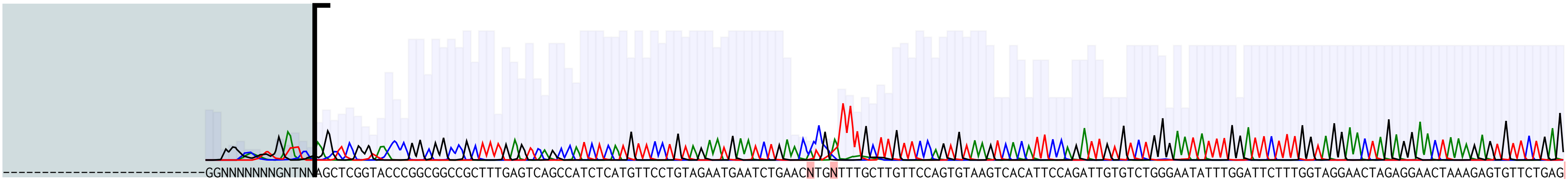

aligned sequence AMY405_PREMIX_AMY405_6 (AMY405_PREMIX_AMY405_6.ab1)

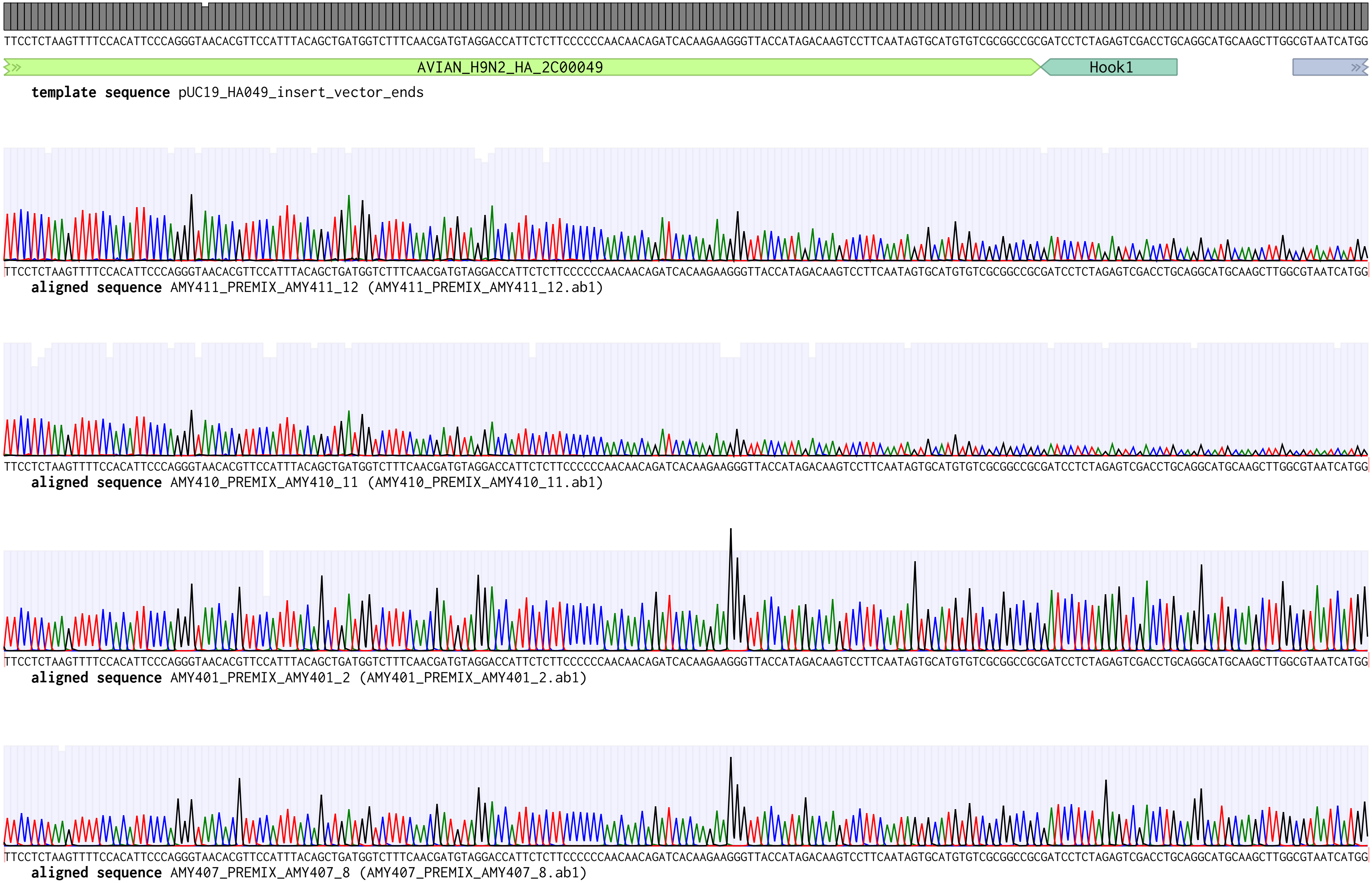
TTCCTCTAAGTTTTCCACATTCCCAGGGTAACACGTTCCATTTACAGCTGATGGTCTTTCAACGATGTAGGACCATTCTCTTCCCCCCAACAACAGATCACAAGAAGGGTTACCATAGACAAGTCCTTCAATAGTGCATGTGTCGCGGCCGCGATCCTCTAGAGTCGACCTGCAGGCATGCAAGCTTGGCGTAATCATGG
AVIAN_H9N2_HA_2C00049
Hook1
template sequence pUC19_HA049_insert_vector_ends
TTCCTCTAAGTTTTCCACATTCCCAGGGTAACACGTTCCATTTACAGCTGATGGTCTTTCAACGATGTAGGACCATTCTCTTCCCCCCAACAACAGATCACAAGAAGGGTTACCATAGACAAGTCCTTCAATAGTGCATGTGTCGCGGCCGCGATCCTCTAGAGTCGACCTGCAGGCATGCAAGCTTGGCGTAATCATGG
aligned sequence AMY411_PREMIX_AMY411_12 (AMY411_PREMIX_AMY411_12.ab1)
TTCCTCTAAGTTTTCCACATTCCCAGGGTAACACGTTCCATTTACAGCTGATGGTCTTTCAACGATGTAGGACCATTCTCTTCCCCCCAACAACAGATCACAAGAAGGGTTACCATAGACAAGTCCTTCAATAGTGCATGTGTCGCGGCCGCGATCCTCTAGAGTCGACCTGCAGGCATGCAAGCTTGGCGTAATCATGG
aligned sequence AMY410_PREMIX_AMY410_11 (AMY410_PREMIX_AMY410_11.ab1)
TTCCTCTAAGTTTTCCACATTCCCAGGGTAACACGTTCCATTTACAGCTGATGGTCTTTCAACGATGTAGGACCATTCTCTTCCCCCCAACAACAGATCACAAGAAGGGTTACCATAGACAAGTCCTTCAATAGTGCATGTGTCGCGGCCGCGATCCTCTAGAGTCGACCTGCAGGCATGCAAGCTTGGCGTAATCATGG
aligned sequence AMY401_PREMIX_AMY401_2 (AMY401_PREMIX_AMY401_2.ab1)
TTCCTCTAAGTTTTCCACATTCCCAGGGTAACACGTTCCATTTACAGCTGATGGTCTTTCAACGATGTAGGACCATTCTCTTCCCCCCAACAACAGATCACAAGAAGGGTTACCATAGACAAGTCCTTCAATAGTGCATGTGTCGCGGCCGCGATCCTCTAGAGTCGACCTGCAGGCATGCAAGCTTGGCGTAATCATGG
aligned sequence AMY407_PREMIX_AMY407_8 (AMY407_PREMIX_AMY407_8.ab1)

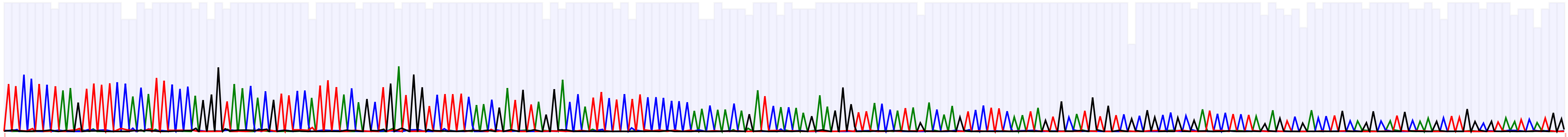

TTCCTCTAAGTTTTCCACATTCCCAGGGTAACACGTTCCATTTACAGCTGATGGTCTTTCAACGATGTAGGACCATTCTCTTCCCCCCAACAACAGATCACAAGAAGGGTTACCATAGACAAGTCCTTCAATAGTGCATGTGTCGCGGCCGCGATCCTCTAGAGTCGACCTGCAGGCATGCAAGCTTGGCGTAATCATGG

aligned sequence ANP862_PREMIX_ANP862_37 (ANP862_PREMIX_ANP862_37.ab1)

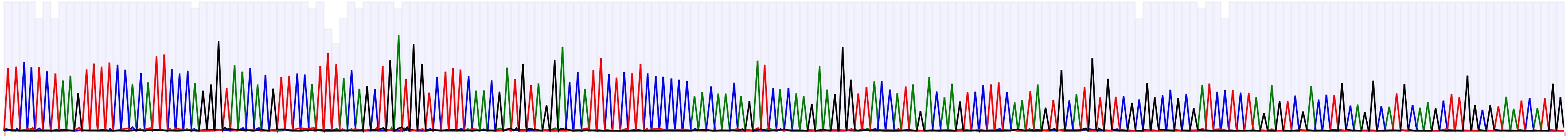

TTCCTCTAAGTTTTCCACATTCCCAGGGTAACACGTTCCATTTACAGCTGATGGTCTTTCAACGATGTAGGACCATTCTCTTCCCCCCAACAACAGATCACAAGAAGGGTTACCATAGACAAGTCCTTCAATAGTGCATGTGTCGCGGCCGCGATCCTCTAGAGTCGACCTGCAGGCATGCAAGCTTGGCGTAATCATGG

aligned sequence AMY403_PREMIX_AMY403_4 (AMY403_PREMIX_AMY403_4.ab1)

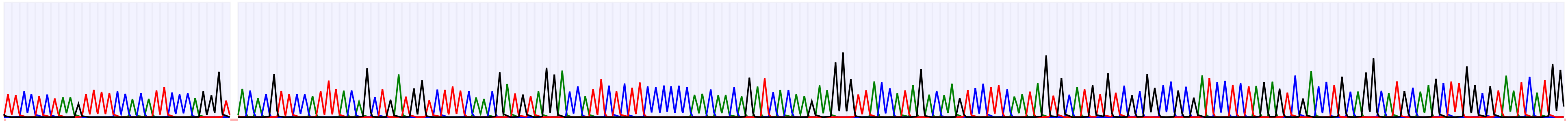

TTCCTCTAAGTTTTCCACATTCCCAGGGT-ACACGTTCCATTTACAGCTGATGGTCTTTCAACGATGTAGGACCATTCTCTTCCCCCCAACAACAGATCACAAGAAGGGTTACCATAGACAAGTCCTTCAATAGTGCATGTGTCGCGGCCGCGATCCTCTAGAGTCGACCTGCAGGCATGCAAGCTTGGCGTAATCATGG

aligned sequence AMY405_PREMIX_AMY405_6 (AMY405_PREMIX_AMY405_6.ab1)
